# A Comparative Transcriptome Analysis of Human and Porcine Choroid Plexus Cells in Response to *Streptococcus suis* Serotype 2 Infection Points to a Role of Hypoxia

**DOI:** 10.3389/fcimb.2021.639620

**Published:** 2021-03-08

**Authors:** Alexa N. Lauer, Rene Scholtysik, Andreas Beineke, Christoph Georg Baums, Kristin Klose, Peter Valentin-Weigand, Hiroshi Ishikawa, Horst Schroten, Ludger Klein-Hitpass, Christian Schwerk

**Affiliations:** ^1^ Pediatric Infectious Diseases, Department of Pediatrics, Medical Faculty Mannheim, University of Heidelberg, Mannheim, Germany; ^2^ Institute for Cell Biology, University Hospital Essen, Essen, Germany; ^3^ Institute for Pathology, University of Veterinary Medicine, Hannover, Germany; ^4^ Faculty of Veterinary Medicine, Institute of Bacteriology and Mycology, Leipzig University, Leipzig, Germany; ^5^ Faculty of Veterinary Medicine, Institute of Veterinary Pathology, Leipzig University, Leipzig, Germany; ^6^ Institute for Microbiology, University of Veterinary Medicine, Hannover, Germany; ^7^ Laboratory of Clinical Regenerative Medicine, Department of Neurosurgery, Faculty of Medicine, University of Tsukuba, Tsukuba, Japan

**Keywords:** next generation sequencing, *Streptococcus suis*, meningitis, choroid plexus, host-pathogen interaction, blood-cerebrospinal fluid barrier

## Abstract

*Streptococcus suis* (*S. suis*) is an important opportunistic pathogen, which can cause septicemia and meningitis in pigs and humans. Previous *in vivo* observations in *S. suis*-infected pigs revealed lesions at the choroid plexus (CP). *In vitro* experiments with primary porcine CP epithelial cells (PCPEC) and human CP epithelial papilloma (HIBCPP) cells demonstrated that *S. suis* can invade and traverse the CP epithelium, and that the CP contributes to the inflammatory response *via* cytokine expression. Here, next generation sequencing (RNA-seq) was used to compare global transcriptome profiles of PCPEC and HIBCPP cells challenged with *S. suis* serotype (ST) 2 infected *in vitro*, and of pigs infected *in vivo*. Identified differentially expressed genes (DEGs) were, amongst others, involved in inflammatory responses and hypoxia. The RNA-seq data were validated *via* quantitative PCR of selected DEGs. Employing Gene Set Enrichment Analysis (GSEA), 18, 28, and 21 enriched hallmark gene sets (GSs) were identified for infected HIBCPP cells, PCPEC, and in the CP of pigs suffering from *S. suis* ST2 meningitis, respectively, of which eight GSs overlapped between the three different sample sets. The majority of these GSs are involved in cellular signaling and pathways, immune response, and development, including inflammatory response and hypoxia. In contrast, suppressed GSs observed during *in vitro* and *in vivo S. suis* ST2 infections included those, which were involved in cellular proliferation and metabolic processes. This study suggests that similar cellular processes occur in infected human and porcine CP epithelial cells, especially in terms of inflammatory response.

## Introduction

The Gram-positive bacterium *Streptococcus suis* (*S. suis*) is an important opportunistic zoonotic pathogen, of which serotype 2 (ST2) is the most prevalent among invasive isolates from the natural host, the pig, as well as for humans (Goyette-Desjardins et al., 2014). The most common clinical manifestation for pigs and humans is meningitis, but septicemia, endocarditis, arthritis, pneumonia, and peritonitis are also reported. Noteworthy, *S. suis* is considered as a re-emerging pathogen especially in Asian countries (Lun et al., 2007; Dutkiewicz et al., 2017; Dutkiewicz et al., 2018).

Previous observations made in natural and experimentally *in vivo* infected pigs revealed that *S. suis* infections are associated with lesions at the choroid plexus (CP) (Sanford, 1987; Williams and Blakemore, 1990; Beineke et al., 2008). The CP forms an interface between the blood and the central nervous system (CNS), the so-called blood-cerebrospinal fluid barrier (BCSFB). The main barrier property at the CP results from the polarized epithelial cells, which are connected *via* tight junctions and tight junction-associated proteins, such as ZO1 and occludin (Wolburg et al., 2001). Experiments performed *in vitro* using primary porcine CP epithelial cells (PCPEC) or immortal human CP epithelial papilloma (HIBCPP) cells demonstrated that *S. suis* can invade and traverse the CP epithelial cells. As a consequence, challenging the CP with *S. suis* contributes to the immunological response *via* cytokine and chemokine expression and secretion (Tenenbaum et al., 2008; Schwerk et al., 2011; Schwerk et al., 2012).

Some efforts have been made to investigate the response of host cells and organisms to infection with *S. suis*. Previous *in vitro* studies involving infection of different cell types with *S. suis*, such as human brain microvascular endothelial cells (HBMEC) or immune cells, including THP-1 monocytes, J774 macrophages, and dendritic cells, have focused on host-pathogen interaction, the inflammatory response and intracellular signaling of the host cells, or on bacterial survival and their interaction with host cells (Charland et al., 2000; Segura et al., 2002; Segura et al., 2004; Meijerink et al., 2012). Whole transcriptome analyses on host cells after challenge with *S. suis* have been performed using THP-1 monocytes, porcine alveolar macrophages, and primary porcine choroid plexus epithelial cells (PCPEC) (De Greeff et al., 2010; Liu et al., 2011b; Schwerk et al., 2011). Differentially expressed genes (DEGs) identified during these studies belonged to different functional categories. Amongst others, genes involved in immune response and host defense, apoptosis/programmed cell death, and those involved in signal transduction pathways were found to be overrepresented (De Greeff et al., 2010; Liu et al., 2011b; Schwerk et al., 2011).

Approaches to investigate the host response to *S. suis in vivo* have been performed in several organisms, including pigs (Li et al., 2010; Liu et al., 2011a; Gaur et al., 2014; Ye et al., 2017), but also non-porcine species, such as zebrafish and mice (Wu et al., 2010; Rong et al., 2012). Confirming the results of *in vitro* challenged host cells, during these studies genes involved in inflammatory and immune defense responses were identified as DEGs (Li et al., 2010; Liu et al., 2011a; Rong et al., 2012; Ye et al., 2017). Tissues and organs investigated during the porcine *in vivo* studies covered spleen, brain, lung, monocytes, and peritoneal blood, but not specifically the CP. Therefore, the transcriptomic changes at the CP of pigs suffering from meningitis after infection with *S. suis* are unknown, leaving participating target genes and host signaling pathways open for discovery. Furthermore, a comparative analysis of the transcriptional response at the CP, either after infection *in vitro vs. in vivo*, or between human and porcine model systems, has not yet been performed. Due to the function of the CP as entry gate into the CNS during infection with *S. suis*, and its role during inflammatory responses to the pathogen, transcriptome analyses of infected pigs and CP epithelial cell cultures will reveal important differently regulated genes that play a role during these processes and point to the cellular pathways involved.

In this study, we employed next generation sequencing (RNA-seq) to compare changes in global transcriptome profiles caused by *S. suis* SS2 at the CP of infected pigs *in vivo*, as well as in PCPEC and HIBCPP cells infected *in vitro*. Identified DEGs and enriched hallmark gene sets (GSs) point to involvement of cellular signaling, immune and inflammatory responses, and hypoxia, whereas cellular proliferation and metabolic processes are rather suppressed during infection. Additionally, the inflammatory responses appear to involve similar cellular processes in infected human and porcine CP epithelial cells.

## Materials and Methods

### Ethics Statement

*In vivo* piglet infection experiments, and the subsequent necropsy, were carried out by veterinarians, in compliance with the principles outlined in the European Convention for the Protection of Vertebrate Animals Used for Experimental and Other Scientific Purposes, as well as the German Animal Protection Law (Tierschutzgesetz). The CP tissue samples analyzed in this study originated from two different *in vivo* infection studies. The CP tissue samples from the meningitis-free animals were part of a study, which was approved by the Landesdirektion Sachsen, with the permit number TVV28/16, which includes approval through the registered committee for animal experiments. The experiment analyzing the CP from animals suffering from meningitis were approved by the Committee on Animal Experiments of the Lower Saxonian State Office for Consumer Protection and Food Safety under the permit number 33.12-42,502-04-16/2305A (Rungelrath et al., 2018). The piglets of both studies originated from the same German Landrace herd that is based on the genotyping results of more than 400 *S. suis* isolates free of the *S. suis* pathotype investigated in this study.

### *In Vivo* Porcine Infection Experiments

The CP tissue samples used for the transcriptome analysis were collected from two independent *in vivo* porcine infection experiments using approximately 8-week-old German Landrace pigs. The animals, from which “meningitis-free” samples were obtained, were three 8-week-old male piglets intravenously infected with 2 × 10^8^ colony forming units (CFU) of *S. suis* ST7 strain 13-00283-02 in the 5th week of life within an unpublished experimental infection. The three piglets, from which “meningitis” samples were obtained, were experimentally infected with *S. suis* ST2 strain 10. Experimental infection was conducted in anesthesia of 8-week-old male piglets *via* intranasal application of 1.5 × 10^9^ CFU following a treatment with 1% acetic acid of the mucosal surface of the nasal cavity as part of a published study carried out by Rungelrath and colleagues (Rungelrath et al., 2018).

In both experiments, the animals were monitored and examined every 8 h post-infection for the onset of disease and were given a clinical score, based on their body temperature, food uptake, and overall behavior (alertness, breathing pattern, mobility). An animal was considered morbid if it presented with a fever of at least 40.2°C. If a high fever persisted (at least 40.5°C), along with apathy and/or anorexia over a 36 h time period, the piglet was euthanized for predefined animal welfare reasons. Additionally, if the piglet presented severe clinical signs, such as opisthotonus, convulsions, inability to rise, or acute polyarthritis, the animal was subject for immediate euthanasia.

Onset of severe disease usually occurred 3 to 5 days post-infection. Once a predefined humane endpoint was reached, the animal was anesthesised through application of 2 mg azaperone (Stresnil^®^, Firma Yanssen) and 10 mg ketamine hydrochloride (Ursotamin^®^, Serumwerke Bernburg) per kg body weight intramuscularly and subsequently euthanized *via* the intravenous administration of T61^®^ (200 mg ml^−1^ embutramide, 50 mg ml^−1^ mebezonium, and 5 mg ml^−1^ tetracain; Intervet, Merck, Sharp & Dohme). The “meningitis-free” piglets were euthanized the same way after observation of 23 days following the experimental ST7 infection.

Immediately following the euthanasia of each animal, a necropsy was performed, which has been previously described by Baums and colleagues (Baums et al., 2006). Additionally, the brain was promptly removed and cut between the two brain hemispheres in order to gain access to the lateral ventricles. One of the two CP from the lateral ventricles was removed, briefly rinsed in sterile DPBS, and flash-frozen in liquid nitrogen for subsequent RNA isolation and transcriptome analysis.

### Cell Culture

Human choroid plexus papilloma (HIBCPP) cells have been described previously as *in vitro* model of the BCSFB (Schwerk et al., 2012). HIBCPP cells were cultivated in DMEM/F12 (Ham) medium with 4 mM L-glutamine and 15 mM HEPES, supplemented with 100 U/ml penicillin, 100 U/ml streptomycin, and 5 μg/ml insulin (HIBCPP medium) and containing 15% FCS. For infection assays cells were seeded on ThinCert™ cell culture filter membranes (Greiner Bio-One, Frickenhausen, Germany), with a 3 μm pore diameter, a pore density of 2.0 × 10^6^ pores per cm^2^, and a membrane diameter of 0.33 cm^2^, in the inverted BCSFB model and maintained as previously described (Schwerk et al., 2012; Dinner et al., 2016), with some modifications. Briefly, a maximum of 100,000 cells were seeded on the membrane of inverted cell culture filter inserts, which, when confluent, resulted in approximately 400,000 cells per filter. HIBCPP cells up to passage 38 were used for experiments. The following day filter membranes were flipped, and placed hanging into a 24-well cell culture plate. The cells were supplied with HIBCPP cell culture medium containing 10% FCS that was exchanged every second day. Approximately 3 to 4 days post-seeding TEER values were monitored with an epithelial tissue voltohmmeter (Millipore, Schwalbach, Germany). Once a TEER of at least 70 Ω x cm^2^ was reached, cells were transferred to HIBCPP cell medium containing 1% FCS. For infection experiments, filters with a TEER of at least 240 Ω x cm^2^ and a maximum of 740 Ω x cm^2^ were used, which was reached 1 or 2 days later.

The isolation and culture of primary PCPEC was performed as previously described (Gath et al., 1997; Haselbach et al., 2001) with some modifications. Briefly, CP tissue was collected from the lateral ventricle of freshly slaughtered pigs at the meat-processing center, and subjected to treatment with 0.2% trypsin (Biochrom, Berlin, Germany, 45 min at 4°C, 17 min at 37°C). Following this cold/warm trypsin treatment, 1 volume of pre-warmed FCS was added before the cell suspension (undigested CP tissue was removed) was centrifuged for 10 min at 55 × g at room temperature, and the pellet was resuspended in 10 ml DMEM (1×) with GlutaMAX™ (Gibco Life Technologies, Paisley, UK) with 4.5 g L^−1^ D-Glucose, pyruvate, and phenol red, 2% (v/v) P/S, and 0.05% (v/v) human recombinant insulin solution (Sigma-Aldritch, Steinheim, Germany) (PCPEC medium) containing 10% FCS and 20 µM Cytosine Arabinoside (AraC, Cell Pharm GmbH, Hannover, Germany). Subsequently, 100 μl of the cell suspension was seeded on the membrane of inverted ThinCert™ cell culture filter inserts (Greiner Bio-One, Frickenhausen, Germany), which were pre-coated with mouse laminin. The following day the filters were washed once by flipping and hanging the cell culture filter membranes into a 24-well cell culture plate containing pre-warmed DPBS (containing Ca^2+^ and Mg^2+^) in order to remove non-adherent cells. Cells were then cultivated for 4 days in PCPEC medium containing 10% FCS and 20 µM AraC, followed by cell culture in AraC-free medium with a medium change every 2 days. Starting 6 days post-seeding, the TEER development was monitored with an epithelial tissue voltohmmeter (Millipore, Schwalbach, Germany). Once the TEER reached approximately 100 Ω x cm^2^, the cells were transferred to PCPEC medium without FCS. A confluent primary PCPEC layer on the 24-well cell culture filter membrane consisted of approximately 60,000 cells. For infection experiments filters with a TEER of at least 250 Ω x cm^2^ were used.

### Measurement of Barrier Integrity

In order to evaluate the barrier development and barrier integrity of HIBCPP cells and PCPEC, the TEER was measured by utilizing a voltohmmeter coupled with a chopstick STX01 electrode (Millipore, Schwalbach, Germany). To calculate the resistance across the cell culture filter membrane area (Ω x cm^2^), the measured Ω, from which the blank value was subtracted (cell culture membrane filter not containing any cells) was multiplied by the surface area of the 24-well cell culture filter membrane (approximately 0.336 cm^2^).

In order to monitor the paracellular permeability of HIBCPP cells and PCPEC throughout the infection intervals, the permeability for FITC-coupled inulin was determined as previously described (Schwerk et al., 2012).

### Bacterial Strains and Cultivation

*S. suis* ST2 strain 10, kindly provided by Hilde Smith (Lelystad, NL), has been used in various studies for experimental induction of disease including meningitis (Vecht et al., 1992; Smith et al., 1999). *S. suis* ST7 strain 13-00283-02 belongs to sequence type 29 and has recently been characterized (Rieckmann et al., 2018). The genomes of both strains have been sequenced (Bunk et al., 2021). Bacteria were cultivated in liquid Todd Hewitt Broth (THB; Oxoid, Wesel, Germany) in order to prepare bacteria stocks for long-term storage and infection experiments. The medium for long-term preservation at −80°C was composed of bacteria suspended in DMEM/F-12 (1×) medium (containing L-Glutamine, 15 mM HEPES, without phenol red; Gibco/Life Technologies™, Paisley, UK) with an optical density at 600 nm (OD_600_) of 0.65, containing 20% glycerol (Sigma-Aldrich, Steinheim, Germany).

In order to cultivate bacteria for the infection experiments, long-term storage stock aliquots were added to 10 ml Todd Hewitt Broth (THB) and incubated at 37°C to mid-log phase. Subsequently, bacterial cultures were washed twice with the appropriate medium used during the infection experiment (see above). The bacterial cell suspension was adjusted to an OD_600_ of 0.65, which resulted in approximately 2 × 10^8^ CFU ml^−1^.

In order to determine the bacterial CFU, which was used for infection of host cells *in vitro*, as well as the CFU throughout the infection time points, the inoculum and an amount, which equaled a multiplicity of infection (MOI) of 10, was serial diluted and plated in duplicate onto Colombia sheep blood agar plates (Oxoid, Wesel, Germany). The sheep blood agar plates were incubated inverted in a humidified incubator at 37°C and 5% CO_2_ overnight, and the CFU were counted the following day.

### *In Vitro* Infection Experiments

HIBCPP cells and PCPEC were infected *in vitro* with *S. suis* ST2 strain 10 once the TEER values reached at least 250 Ω×cm^2^. Infection was performed with a MOI of 10. The HIBCPP cells were infected up to 10 h, with time points being in 2 h increments. The primary PCPEC were infected up to 6 h, again, with time increments being in 2 h. Throughout the infection time points, TEER values and paracellular permeability were determined to evaluate barrier integrity.

### Cell Viability—Live/Dead Assay

To determine the cell viability of PCPEC and HIBCPP cells live/dead-assays (Invitrogen, Karlsruhe, Germany) were performed according to the manufacturer’s instructions. In this assay living cells appear green (intracellular esterase activity), whereas dead cells appear red (loss of plasma membrane integrity allows entry and DNA binding of ethidium homodimer-1). Results were documented by immunofluorescence microscopy.

### RNA Isolation

The RNeasy Mini Kit (Qiagen, Hilden, Germany) was used for total RNA isolation from HIBCPP cells and the CP tissue from the *in vivo* porcine experiments. For the HIBCPP cell infection experiments, filters were briefly rinsed with DPBS (with Ca^2+^ and Mg^2+^), before the cells were processed following the manufacturers protocol “purification of total RNA from animal cells using spin technology.” For the CP tissue collected from the *in vivo* porcine infection experiments, 30 mg of fresh-frozen CP tissue was suspended in 600 μl lysis buffer and homogenized using a micro pestle. Subsequently, the homogenized tissue suspension was applied to a QiaShredder column (Qiagen, Hilden, Germany) before RNA was isolated according to the manufacturer’s protocol “purification of total RNA from animal tissues.” The RNeasy Micro Kit (Qiagen, Hilden, Germany) was used for total RNA isolation from PCPEC. Lysates from four cell culture membranes were pooled. The manufacturer’s protocol “purification of total RNA from animal and human cells” was followed. All isolated RNA samples underwent an on-column DNase I treatment (15 min incubation at room temperature), which was integrated during the RNA isolation protocols, as described by the manufacturer (Qiagen, Hilden, Germany). Following the isolation, RNA samples were eluted in RNase-free H_2_O and stored at −80°C. The RNA concentration was determined with a spectrophotometer (ND1000, Peqlab Biotechnoloy, Erlangen, Germany).

### Semiquantitative RT-PCR and QPCR

Total RNA (0.5 μg RNA from HIBCPP cells and from the CP tissue from the *in vivo* porcine infection experiments; 0.125 μg from PCPEC) was reverse transcribed using random primers included in the AffinityScript QPCR cDNA Synthesis Kit (Agilent Technologies). Semi-quantitative PCR reactions applying defined volumes of the generated cDNA were performed with the Taq DNA Polymerase Kit (Qiagen) following the manufacturers’ instructions. PCR reaction mixtures were initially denatured for 2 min at 94°C and subsequently subjected to the indicated cycles of denaturation (94°C, 30 s), annealing (55–60°C, depending on the primers, 30 s), and extension (72°C, 1 min) followed by a final extension step at 72°C for 7 min. PCR products were visualized by gel electrophoresis using 1.5% agarose gels and ethidium bromide staining.

To quantitatively evaluate the expression of selected genes, the Brilliant II SYBR^®^ Green QPCR Master Mix kit (Agilent Technologies) was used according to the manufacturer’s instructions. The qPCR was run using the Stratagene Mx3005P system with the MX software using the one plateau pre-melt/RT segment and normal two-step amplification setting, followed by determination of a dissociation curve. The following conditions were applied: initial denaturation (95°C, 10 min) followed by 40 cycles of denaturation (95°C, 30 s), combined annealing and extension (58°C, 60 s), and the denaturation curve (95°C, 60 s; 65°C, 30 s; 95°C, 30 s). Fold-changes were calculated using the 2^−ΔΔCt^ method (Livak and Schmittgen, 2001) and expression of the gene for Glyceraldehyde 3-phosphate dehydrogenase (GAPDH) was used as control.

Primers for RT-PCR and QPCR were designed employing Primer3 software (Rozen and Skaletsky, 2000) and are shown in **Supplementary Tables S1** and **S2**, respectively. The primer sequences were synthesized by Sigma-Aldrich (Steinheim, Germany), delivered in a lypophilic form, and reconstituted in double-distilled water.

### RNA-Seq Analyses

RNA samples used for RNA-seq analysis were evaluated for their RNA integrity with the Agilent 2100 Bioanalyzer System (Agilent Technologies, Waldbronn, Germany) in combination with the Agilent Bioanalyzer RNA Nano Chip. RNA integrity numbers were above 9.5 for RNA isolated from HIBCPP cells, above 8.9 for RNA isolated from PCPEC, and above 7.4 for RNA isolated from CP tissues obtained during the *in vivo* porcine infection experiments.

The Ovation^®^ Human FFPE RNA-Seq Multiplex System Kit (NuGen, Illumina, San Diego, CA, USA) was utilized for library preparation from 100 ng of total RNA from the HIBCPP cells for conventional RNA-seq. For the PCPEC and the porcine CP tissues from the *in vivo* infection experiments, the Universal Plus mRNA-Seq kit (NuGen, Illumina, San Diego, CA, USA) was used for the RNA library preparation from 100 ng total RNA.

For transcript fragmentation the Covaris S220 Focused-ultrasonicator, with the Covaris microTUBE, set to 10% duration, 200 cycles per burst, and 140 s was used to generate fragment sizes of approximately 250 bp long. Fragment lengths were analyzed on the Agilent 2100 Bioanalyzer System using the Agilent Bioanalyzer HS DNA chip. Bead-purification steps were carried out with the help of Agencourt AMPure XP SPRI beads in order to remove adapter or primer dimers throughout the various cDNA synthesis, adaptor ligation, or PCR amplification steps. The Qubit system was used in combination with the double stranded DNA high sensitivity assay kit in order to determine the DNA concentration of the prepared libraries before these were pooled. Adaptor ligation introduces specific 8 bp long barcode sequence to each of the individual biological replicates of infected and uninfected samples. With the use of the barcodes, one library pool containing all the biological replicates for each phenotype (infected and uninfected) was generated for sequencing. The library pool was quantified utilizing the NEBNext^®^ Library Quant kit, which is a SYBR Green based qPCR method essential in order to achieve the optimal cluster density on the sequencing flow cell required for an optimal sequencing output.

The sequencing of the pooled HIBCPP cell sample set library was performed using HiSeq 2500 flow cell system (Illumina^®^, San Diego, CA, USA) in the high-output and paired-end mode. The sequencing of the pooled RNA libraries of the PCPEC samples and the *in vivo* CP samples was carried out on the HiSeq 2500 flow cell system (Illumina^®^, San Diego, CA, USA) in the rapid-run and paired-end mode, sequencing 100 bp length for each read.

### Bioinformatic Processing Post-Sequencing

Subsequently to the sequencing process, the generated raw reads were processed. In a first step the adapter sequences were removed with the help of the Trimmomatic-0.30 software tool, which was designed to identify full and partial adapter sequences from single- and paired-end Illumina NGS data (Bolger et al., 2014). The trimmed sequences were subject to alignment by utilizing the RNA aligner STAR software (Dobin et al., 2013). The sequences generated from the HIBCPP cells were aligned to the human genome 19 and the sequences generated from the porcine samples (PCPEC and CP tissue from the *in vivo* infection experiments) were aligned to the *Sus scrofa* genome 11.1. The aligned sequences of the HIBCPP cell and PCPEC samples were imported into the StrandNGS software and filtered on quality metrics, which included the removal of reads that had more than one match, an alignment score of below 95, mapping quality below 40, and lengths less than 20. This additional filtering step was not carried out on the sequences aligned from the *in vivo* experiment. Instead, these sequences were subject to Universal Molecular Identifiers filtering, which resulted in less than 5% of reads removed. These aligned and filtered reads can be imported to the Integrative Genomics Viewer, which allows visualization of the NGS datasets (Robinson et al., 2011).

Lastly, for the quantification of the RNA sequences, the aligned and filtered reads were imported to the Partek Genomics Suite (PartekGS) software. This software reports the reads per kilobase of exon model per million reads (RPKM), which is a scaling method applied in order to normalize the RNA abundancies for sequencing depth (library size) and gene length (Mortazavi et al., 2008).

### RNA-Seq Statistical Data Analysis: DEGs and GSEA

In order to evaluate the differential gene expression and to utilize data analysis platforms for downstream analysis, RPKM values and/or the raw reads were used to generate the Differentially Expressed Genes (DEGs) list and to perform a Gene Set Enrichment Analysis (GSEA). DEGs were identified using the PartekGS software implementing the one-way analysis of variance (ANOVA) test. A step-up method to correct for multiple testing was applied to generate corrected *p*-values, but due to the low number of replicates, the correction for multiple testing eliminated many potential true-positive targets. For this reason, the identification of DEGs was based on uncorrected *p*-values. The resulting *p*-values were exported and further manual filtering steps were carried out in Excel from Microsoft Office. Filters, which were applied in order to generate the final DEG list, included at least 10 (for the PCPEC and *in vivo* samples) or 20 (for the HIBCPP cell samples) raw reads of the transcript in the biological triplicates of one class (uninfected versus infected) and an uncorrected *p*-value of ≤0.05. Additionally, a more stringent list was created which only displayed a list of significant DEGs with a corrected (step-up method) *p*-value ≤0.05 (De and Baron, 2012). The genes resulting in the final DEG list with an uncorrected and corrected *p*-value ≤0.05 were taken into consideration.

A second statistical analysis was applied using the RPKM values, by utilizing the GSEA software, which allows the user to interpret the data on a biological relevant gene set level, and not on individual genes, when comparing different biological states (Mootha et al., 2003; Subramanian et al., 2005). The GSEA makes use of the Molecular Signature Database (MSigDB), which contains approximately 18,000 gene sets (GSs), categorized into eight major groups (Subramanian et al., 2005; Liberzon et al., 2011; Liberzon et al., 2015). The GSEA was run with the standard settings in the gene set permutation mode, set to 1,000 permutations. In order to select potential interesting candidates, GSs which presented a false discovery rate (FDR) *q*-value ≤0.25 were considered to be of interest, along with a normalized enrichment score (NES) of approximately ±2.

### Accession Numbers

The data generated during RNA-seq was deposited in the Sequence Read Archive (SRA) on the National Center for Biotechnology Information (NCBI) platform. The BioProject accession number for the HIBCPP cell data is PRJNA533919. The BioProject accession number for the PCPEC data is PRJNA533792. The BioProject accession number for the data acquired from the CP tissue which was isolated following the *in vivo* infection experiments and sequenced *via* conventional RNA-seq is PRJNA534398.

### Statistics

The significance of the gene fold change from infected samples in comparison to uninfected samples was calculated using the unpaired student’s t-test. A result was considered significant if *p* ≤ 0.05.

## Results

### Infection of HIBCPP Cells and PCPEC With *S. suis* ST2

**Figure 1:**
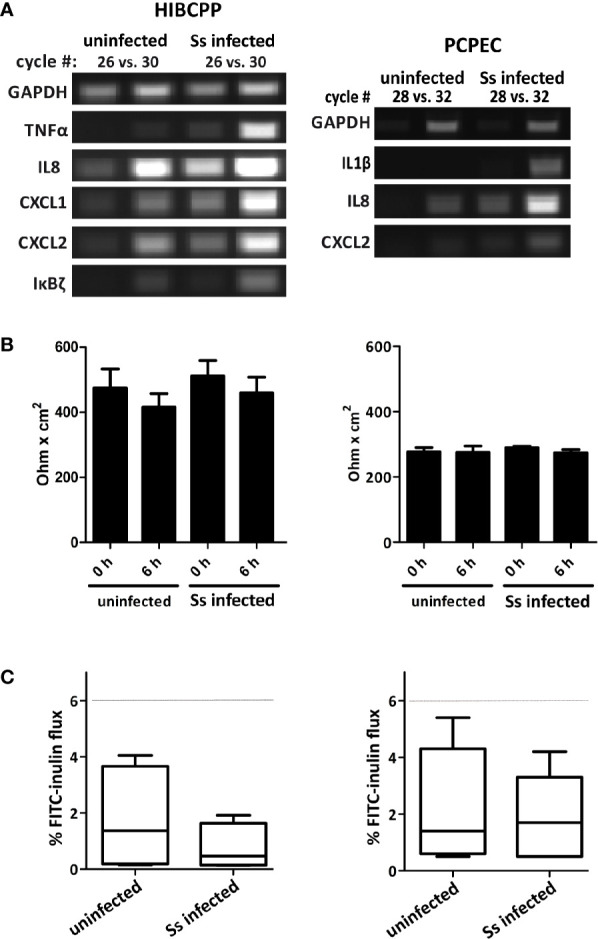
Infection of HIBCPP cells (left) and PCPEC (right) with *S. suis* ST2 (indicated as Ss) causes induction of an inflammatory response. **(A)** HIBCPP cells and PCPEC grown on inverted cell culture filter inserts were treated with *S. suis* ST2 at a MOI of 10 for 6 h and semi-quantitative RT-PCR was performed to determine induction of the genes indicated at the left of the panels. PCR reactions were analyzed after the cycle numbers indicated at the top of the panels. Uninfected HIBCPP cells and PCPEC served as controls **(B)** TEER values of uninfected control cells and of infected HIBCPP cells and PCPEC were measured at the beginning of the experiments (0 h) and after 6 h of infection (6 h). **(C)** The flux of FITC-labelled inulin across uninfected control cells and across infected HIBCPP cells and PCPEC was measured after 6 h of infection and stayed below 6% (dotted lines) under all conditions.

We have previously shown that *S. suis* ST2 invades both HIBCPP cells and PCPEC in a polar fashion from the basolateral side and that the CP contributes to the inflammatory response when challenged with *S. suis* (Tenenbaum et al., 2009; Schwerk et al., 2011; Schwerk et al., 2012). To determine time points following infection of HIBCPP cells and PCPEC, at which the cells demonstrate a transcriptional response, we treated cells grown on inverted cell culture filter inserts with *S. suis* ST2 for different amounts of time and analyzed the expression of selected inflammatory response genes by semi-quantitative RT-PCR (**Figure 1** and data not shown). As shown in **Figure 1A** both HIBCPP cells and PCPEC displayed a strong transcriptional response after 6 h of challenge with *S. suis* ST2. Importantly, the barrier function of both HIBCPP cells and PCPEC stayed intact during this time period as indicated by a stable TEER (**Figure 1B**) and low permeability by FITC-labelled inulin (**Figure 1C**). Furthermore, live/dead assays demonstrated that both cell types remained viable during the infection period (data not shown). Therefore, we selected HIBCPP cells and PCPEC infected with *S. suis* ST2 for 6 h in the inverted cell culture insert model for further transcriptome analyses.

### The CP of In Vivo Infected Piglets Suffering From Meningitis Exhibited an Inflammatory Response

*In vivo* infection experiments with *S. suis* were carried out with 8-week-old piglets. Three meningitis-free control animals intravenously infected with *S. suis* ST7 3 weeks prior euthanasia and three animals suffering from meningitis after intranasal infection with *S. suis* ST2 strain 10 (Rungelrath et al., 2018) were selected for further analysis.

The meningitis-free animals exhibited no clinical signs of central nervous system dysfunction within the entire observation period. Following the conclusion of the experiment, the animals were euthanized and a necropsy was performed. Part of the necropsy consisted of CSF, swabs, and tissue collections for bacteriological investigations and preserving respective predefined tissues in formalin for histopathological evaluation as described elsewhere (Baums et al., 2006; Rungelrath et al., 2018). No bacterial growth was observed on Columbia blood agar plates used for cultivation of CSF and brain swabs. Furthermore, the absence of fibrinous and purulent inflammations in the brain, meninges and the CP was confirmed for the three animals included in this study. These results are summarized in **Table 1**.

**Table 1:** Summary of the clinical signs, the specific bacterial loads of CSF, and the histopathological lesions in the brain found in piglets post-infection with *S. suis* ST7 (piglets 17, 24, and 43) and ST2 (piglets 4, 5, and 34).

Animal Number	Symptoms post-infection	Bacterial CFU per ml of CSF	Histopathological findings
**Meningitis-free animals^1^**			
17	Day 1: 40.4°C fever Day 2: no fever	None	No fibrinous or purulent lesion in the brain, meninges, or CP
24	Day 1: 41.1°C then 40.4°C fever Day 2: 40.7°C fever, which cleared by the end of the day	None	No fibrinous or purulent lesion in the brain, meninges, or CP
43	Day 1: 41.1°C then 41.3°C Day 2: 41.3°C, which cleared by the end of the day Day 14: 40.3°C, which cleared by the end of the day	None	No fibrinous or purulent lesion in the brain, meninges, or CP
**Animals suffering from meningitis^2^**			
4	42.1°C fever, ataxia, kyphosis, body tremors, abdominally reinforced breathing	3.33 × 10^7^	Moderate multifocal fibro-purulent meningitis and moderate multifocal plexus chorioiditis
5	41°C fever, ataxia, body tremors, convulsions	5.43 × 10^7^	High grade multifocal fibro-purulent meningitis and moderate multifocal plexus chorioiditis
34	41.7°C fever, body tremors, abdominal reinforced breathing, nystagmus, opisthotonus	3.03 × 10^7^	Moderate multifocal fibro-purulent meningitis (no comment on the state of the CP)

^1^Piglets were euthanized after observation of 23 days following the experimental ST7 infection.

^2^Piglets were euthanized for predefined animal welfare reasons if a high fever persisted (at least 40.5°C), along with apathy and/or anorexia over a 36 h time period or if severe clinical signs, such as opisthotonus, convulsions, inability to rise, or acute polyarthritis were presented.

The onset of clinical signs in the animals infected with *S. suis* ST2 occurred 2 to 4 days post-infection, and included high fever, body tremors, convulsions, and ataxia. Due to the severeness and character of these clinical signs these piglets reached the humane endpoint were euthanized. As with the meningitis-free animals, part of the necropsy consisted of CSF, swab and tissue collection and preserving the tissues in formalin for histopathological evaluation. The CSF of all three animals showed a high amount of bacterial CFUs per ml of CSF, which were confirmed *via* MP-PCR (Silva et al., 2006) to be *mrp*+ *epf*+ *sly*+ *cps*2+ *S. suis* (data not shown). The two piglets (5, 34) which exhibited specific central nervous system dysfunctions and piglet 4 with high fever and tremors were found post-mortem to have a fibro-purulent meningitis *via* histopathological evaluation of the brain and CP as summarized in **Table 1**.

**Figure 2:**
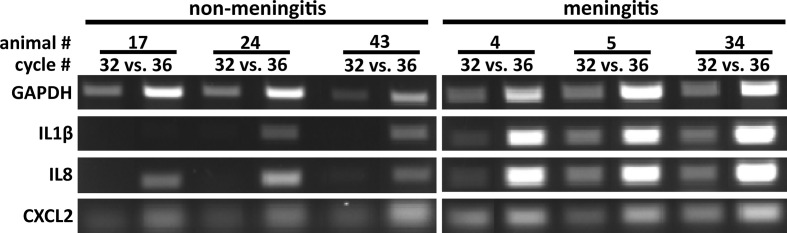
The choroid plexus of *S. suis* ST2 *in vivo* infected piglets shows an inflammatory response in comparison to piglets not suffering from meningitis. Semi-quantitative RT-PCR was performed to determine induction of the genes indicated at the left of the panels and PCR reactions were analyzed after the cycle numbers indicated at the top of the panels. Animals 17, 24, and 43 were meningitis-free, whereas animals 4, 5, and 34 suffered from meningitis.

RNA isolation was performed from one CP of the lateral ventricles in the selected animals. A semi-quantitative RT-PCR was performed in order to confirm an inflammatory response at the CP of *in vivo S. suis* ST2 infected piglets compared to meningitis-free pigs. As depicted in **Figure 2**, the CP isolated from piglets suffering from meningitis showed a clear inflammatory response by the increased expression (enhanced signal when referencing the GAPDH signal) of the genes encoding IL1β and IL8, as compared to the meningitis-free piglets. The signal for the CXCL2 expression, in contrast, was only enhanced for some piglets suffering from meningitis. GAPDH was used as a house-keeping gene, which displayed a non-coherent expression likely due to either the heterogeneous cell population found in the CP tissue or lower RNA integrities compared to RNA from the *in vitro* infection experiments (**Figure 2**).

### Bioinformatic Processing of RNA-Seq Data Revealed Efficient Mapping to Exon Gene Regions

The RNA samples from *S. suis* ST2 challenged HIBCPP cells and PCPEC and control cells as well as from CPs isolated from meningitis-free pigs or from pigs suffering from meningitis were subjected to RNA-seq as described in *Materials and Methods*. Following the sequence run, the generated reads were aligned to either the human or the porcine reference genome with the RNA aligner STAR (Dobin et al., 2013) and quantified in PartekGS.

In total, approximately 20 million reads were generated per sample of the HIBCPP cell sample set and for the samples originating from the *in vivo* infection experiments, and approximately 10 million reads were generated for the PCPEC samples.

For the HIBCPP cell samples, approximately 62–74% of the aligned reads fully overlapped to an exon region of the reference genome (data not shown). The aligned reads obtained from the samples of porcine origin (*in vitro* and *in vivo* samples) displayed a higher full exon overlap, of approximately 80%.

The quality assurance for the transcript alignment and mapping post-sequencing displayed values within acceptable ranges (data not shown) for all samples which underwent sequencing and alignment and were statistically analyzed in the next step.

### Differentially Expressed Genes (DEGs)

We first determined the magnitude of statistically significant DEGs based on the number of reads detected during the alignment and mapping process. A total of 1,479 significant (p ≤ 0.05) DEGs were identified following the analysis of *S. suis* ST2 infected versus uninfected HIBCPP cells. A total of 63 DEGs were identified, which exhibited at least a 2-fold up- or down-regulation, of which 32 genes displayed an up-regulation and 31 genes displayed a down-regulation. The results of the uncorrected p-values are presented in **Supplementary Table S3**, while the correction of the p-value yielded no significantly DEGs (data not shown). The highest significant up-regulation observed was a 3.6 fold change for the gene encoding for ZFPL36L1. The strongest down-regulation was observed for the microRNA3648 with an 8-fold change. Interestingly, the majority of the genes only exhibited a 2- to 3-fold differential expression following the 6 h infection period with *S. suis* ST2.

For the *in vitro S. suis* ST2 infected PCPEC samples, a total of 430 genes were identified as significantly differentially regulated with an uncorrected p-value ≤0.05, of which 46 genes exhibited an up-regulation of at least 2-fold, and 4 genes a down-regulation of at least 2-fold. The results of the uncorrected p-values are presented in **Supplementary Table S4**, since the correction of the p-value yielded only one significant gene (ACOD1; p = 0.025; data not shown). Interestingly, the *in vitro* infected primary PCPEC displayed a very strong cellular response during the infection with *S. suis* ST2, with a maximum of 134-fold up-regulation for IL1β. A total of 21 genes exhibited an up-regulation of at least 3-fold, with the majority of these genes known to being involved in the inflammatory response and regulation. In contrast, a total of only four genes encoding for VSIG4, DLGAP5, KNL1, and STRC were significantly differentially down-regulated at least 2-fold during *S. suis* ST2 infection. The gene encoding for STRC was the highest significantly down-regulated gene, which exhibited a maximum down-regulation of 3-fold change.

A total of 3,355 DEGs with an uncorrected p-value of ≤0.05 were identified in the CP of pigs suffering from *S. suis* ST2—induced meningitis in relation to meningitis-free pigs. The DEG list of significant genes with a corrected p-value ≤0.05 presented 479 genes, which were at least ±2-fold regulated, of which 171 genes were up-regulated and 308 genes were down-regulated (data not shown). Due to this large number, the significant DEGs with corrected p-values are presented in **Supplementary Table S5**. In total, 30 genes which were significantly differentially regulated exhibited a fold change of at least ±2 with a corrected p-value of ≤0.05. Twelve of the 30 genes displayed an up-regulation and 18 genes displayed a down-regulation. The strongest up-regulation was 11.3-fold for the gene encoding INSM1, whereas the strongest down-regulation was 6.3-fold for the gene encoding for TIMD4. Furthermore, of the 30 genes identified in **Supplementary Table S5**, 13 genes exhibited a fold change ±3.

Only two overlapping significant DEGs were identified in the infected CP epithelial cells, HIBCPP cells and PCPEC, and included transcriptional and immune response regulator (TCIM) and Rho family GTPase 1 (RND1).

### Verification of Selected Genes by qPCR

Following the sequencing analysis, the generated data was validated by verifying the expression levels of selected genes by qPCR. The DEG lists presented in *Differentially Expressed Genes (DEGs)* were used in order to select candidate genes for the validation of the sequencing data. Genes, which are implicated during inflammation [IL1β, IL8, C-X-C ligand 2 chemokine (CXCL2), TNFα] and during regulation of the inflammatory response [NFκB inhibitor alpha (NFκBIA), zinc finger CCCH-type containing 12A (ZC3H12A), TCIM] were selected for the verification. Additionally, since hypoxia is implicated during bacterial infections (Schaffer and Taylor, 2015; Zeitouni et al., 2016; Devraj et al., 2017), genes known to play a key role during hypoxia, or are known to have their expression influenced in a hypoxic environment (hypoxia-inducible factor 1 alpha (HIF1α), vascular endothelial growth factor a (VEGFA), MAX interactor 1 (MXI1), dual specificity phosphatase 2 (DUSP2)), were chosen for validation.

**Table 2** summarizes the fold change and the corresponding significance (uncorrected p-value of a one-way ANOVA test) of the selected genes during the RNA-seq analysis compared to the relative fold change (2^−ΔΔCT^) between infected versus uninfected samples, along with the S.D., of the qPCR results. Overall, the gene fold change determined following the RNA-seq data analysis could be validated with the qPCR method, and the significances determined following the qPCR analysis largely corresponded to the significant fold change determined in the RNA-seq experiment. Genes encoding for the cytokines and chemokines displayed a higher fold change in the PCPEC and in samples from the porcine *in vivo* infection experiments, an observation also made from the DEG lists (*Differentially Expressed Genes (DEGs)*).

**Table 2:** Summary of the fold changes and uncorrected *p*-values of selected genes from the RNA-seq data and the fold changes of infected samples in relation to uninfected samples.

	HIBCPP cells				PCPEC				*In vivo*			
	RNA-seq		QPCR		RNA-seq		QPCR		RNA-seq		QPCR	
	FC	*p*-value	relative 2^−ΔΔCt^	S.D.	FC	*p*-value	relative 2^−ΔΔCt^	S.D.	FC	*p*-value	relative 2^−ΔΔCt^	S.D.
IL1β	−1.1	0.925	2.0	3.4	134.0	0.041	136.4	83.0	2.8	0.127	4.6	15.3
IL8	3.8	0.386	3.8	7.3	22.6	0.006	20.5	12.8	22.0	0.014	7.6	17.7
CXCL2	4.9	0.258	4.7	5.3	13.8	0.017	19.4	5.5	9.9	0.005	13.0	25.1
TNFα	12.1	0.256	12.0	17.1	70.7	0.006	19.8	2.6	1.1	0.909	1.2	0.3
NFκBIA	4.2	0.266	2.6	3.5	3.9	0.016	3.5	1.2	1.3	0.173	1.7	0.4
ZC3H12A	3.3	0.220	3.1	2.4	2.1	0.004	2.0	1.2	2.9	0.107	1.4	0.6
TCIM	3.1	0.025	2.6	4.6	2.7	0.001	2.7	0.4	2.2	0.114	3.1	1.9
HIF1α	1.1	0.732	0.8	0.3	−1.6	0.306	1.0	0.2	−1.3	0.008	1.0	0.1
VEGFA	2.0	0.075	1.6	0.5	1.6	0.006	1.4	0.3	1.4	0.003	1.4	0.2
MXI1	2.5	0.043	2.8	12.1	1.2	0.008	1.0	0.03	1.1	0.416	1.1	0.1
DUSP2	−2.6	0.015	−4.2	2.2	5.2	0.016	1.3	1.5	8.1	0.004	9.7	1.9

The fold changes and p-values of the RNA-seq data were determined by the software PartekGS. The fold change for the qPCR data was calculated via the 2^−ΔΔCT^ method using GAPDH as an internal control, and the relative fold change was determined between infected versus uninfected samples with the S.D. Genes are considered significantly differentially regulated if the p-value ≤0.05. These listed genes were selected for subsequent QPCR validation.

FC, fold change; S.D., standard deviation.

### Identification of Enriched Individual Genes by GSEA

**Figure 3:**
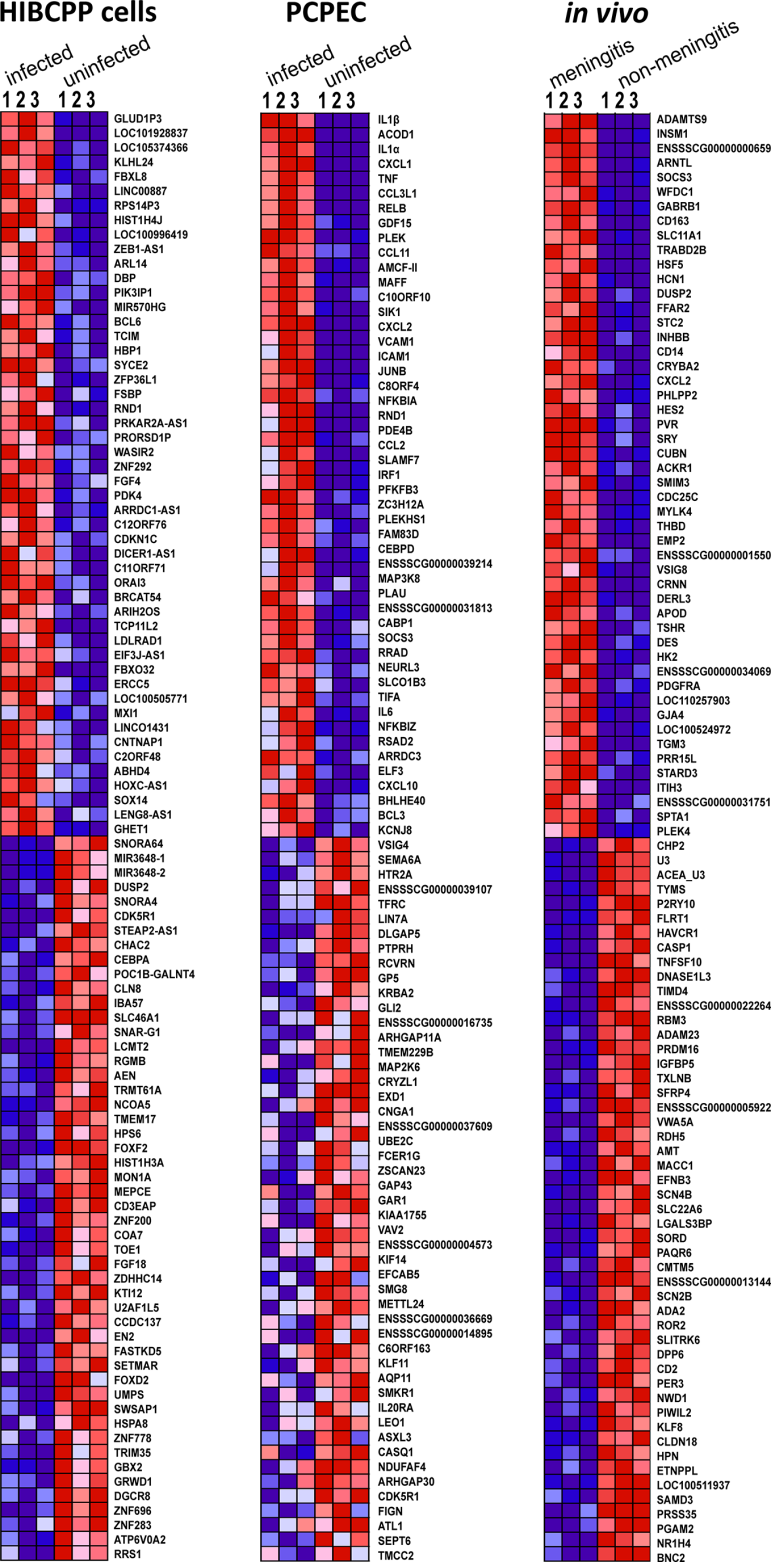
The GSEA generated heat-map depicts a coherent expression of the biological triplicates for all sample sets of the top 50 ranked genes. Dark red indicates high expression, dark blue indicates low expression.

A GSEA was performed in order to evaluate the data in terms of biologically relevant functions. In addition to attributing enriched genes to pre-defined GSs, GSEA also evaluated the individual genes for their enrichment and how well their expression differentiates in the two phenotypes. **Figure 3** depicts the heat-map generated in the GSEA analysis of the top 50 genes, which were found to be most distinguishing between *S. suis* ST2 infected epithelial cells or animals suffering from meningitis and uninfected epithelial cells or meningitis-free animals. The strongest coherent differential expression between the biological triplicates for each phenotype can be observed for the samples, which originated from the porcine *in vivo* experiments. However, strong coherence was observed for the majority of the biological HIBCPP cells and PCPEC replicates. Furthermore, the majority of the GSEA enriched genes were also identified with the PartekGS software, and can be found in the presented DEG lists in *Differentially Expressed Genes (DEGs)*.

Overall, the majority of the top 50 enriched genes displayed coherent differentiation in all biological replicates for the investigated phenotype. Interestingly, the top enriched genes determined by the GSEA software were also identified through the DEG analysis performed with the PartekGS software.

### Identification of Enriched Hallmark GSs by GSEA

The hallmark GS collection consists of 50 GSs, which represent well-defined and coherent biological states, and were created in order to reduce redundancy. Furthermore, these 50 GSs are divided into eight major biological categories of: cellular components, development, immune reaction, cellular metabolism, pathways, cellular proliferation, signaling, and DNA damage (Liberzon et al., 2015).

The GSEA of analyzed HIBCPP cell samples revealed that a total of 18 GSs out of the 50 hallmark GS collection were found to be significantly enriched in 6 h *S. suis* ST2 infected cells. **Table 3** presents these significantly enriched hallmark GSs, which exhibited a FDR q-value of less than or equal to 0.25. The majority of the GSs can be categorized into cell signaling, and include, for e.g., TNFα signaling *via* NFκB, IL2-STAT5, and transforming growth factor beta (TGFβ) signaling. Additionally, five GSs belonging to the immune cellular processes were significantly enriched and include, for e.g., inflammatory response, IL6-JAK/STAT3 signaling, and complement system. Furthermore, the most significantly enriched GS was hypoxia (NES 3.24, q-value 0.000), and is categorized into the cellular pathways, along with apoptosis (NES 1.53, q-value 0.032). The GS epithelial-mesenchymal transition (EMT) was the third significantly enriched GS (NES 1.98, q-value 0.001) and belongs to the category of cellular development.

**Table 3:** GSEA of 18 hallmark GSs, which are significantly enriched in *S. suis* ST2 infected HIBCPP cells.

Hallmark Gene Set Name	Size	NES	FDR q-value
Hypoxia	158	3.24	0.000
TNFα Signaling *via* NFκB	168	2.87	0.000
Epithelial-Mesenchymal transition	112	1.98	0.001
Apical surface	29	1.85	0.002
Glycolysis	171	1.78	0.012
Cholesterol homeostasis	68	1.73	0.012
Inflammatory Response	111	1.69	0.014
IL2-STAT5 Signaling	146	1.60	0.022
Myogenesis	107	1.54	0.030
Apoptosis	126	1.53	0.032
P53 Pathway	177	1.48	0.045
KRAS Signaling_up	124	1.45	0.057
Interferon γ response	159	1.44	0.056
KRAS Signaling_down	70	1.35	0.089
IL6-JAK/STAT3 Signaling	55	1.34	0.090
Allograft rejection	105	1.20	0.210
TGFβ Signaling	48	1.19	0.210
Complement	132	1.19	0.208

The normalized enrichment score (NES) represents the extent of GS enrichment, taking the number of genes (“size”), which compose the GS, into account. The false discovery rate (FDR) is considered significant if q is ≤0.25.

NES, normalized enrichment score; FDR, false discovery rate.

**Table 4** summarizes the enriched GSs of the response of the infected PCPEC. A total of 28 GSs were found to be significantly enriched and displayed a FDR of q ≤ 0.25. The majority of enriched GSs are categorized into the cellular signaling and include the GSs, for e.g., TNFα signaling *via* NFκB, IL2-STAT5, and TGFβ. The category with the second most enriched GSs was found to belong to the immune response and include, for e.g., interferon α, γ, and inflammatory response, IL6-JAK/STAT3 signaling, and complement system. The category containing the third most enriched GSs for the infected or meningitis phenotype was the cellular pathway and included hypoxia, apoptosis, unfolded protein response, and reactive oxygen species pathway. Additionally, the GSs angiogenesis and EMT, which are categorized into cellular development, were found to be enriched.

**Table 4:** GSEA of 28 hallmark GSs, which are significantly enriched in infected primary PCPEC.

Hallmark Gene Set Name	Size	NES	FDR q-value
TNFα signaling *via* NFκB	151	3.12	0.000
Interferon γ response	135	2.49	0.000
Inflammatory response	105	2.48	0.000
Allograft Rejection	108	2.20	0.000
IL6-JAK/STAT3 Signaling	48	2.19	0.000
Hypoxia	146	2.12	0.000
IL2-STAT5 Signaling	129	1.99	0.000
Interferon α response	73	1.90	0.001
Complement	136	1.88	0.001
UV response_up	115	1.87	0.001
Apoptosis	123	1.85	0.002
P53 Pathway	154	1.64	0.016
Coagulation	78	1.54	0.034
Unfolded protein response	93	1.51	0.044
Wnt/β-catenin	31	1.49	0.047
Reactive oxigen species pathway	40	1.47	0.051
Estrogen response_early	136	1.41	0.083
KRAS Signaling_up	127	1.39	0.090
Angiogenesis	23	1.37	0.101
NOTCH Signaling	25	1.36	0.102
KRAS Signaling_down	78	1.28	0.178
TGFβ Signaling	47	1.27	0.177
Xenobiotic Metabolism	129	1.24	0.200
Epithelial-Mesenchymal transition	153	1.24	0.192
Glycolysis	145	1.23	0.195
Cholesterol homeostasis	63	1.23	0.187
Androgen response	86	1.23	0.187
MTORC1 Signaling	162	1.20	0.210

The normalized enrichment score (NES) represents the extent of GS enrichment, taking the number of genes (“size”), which compose the GS, into account. The false discovery rate (FDR) is considered significant if q is ≤0.25.

NES, normalized enrichment score; FDR, false discovery rate.

**Table 5** summarizes the enriched GSs of the response of the CP in animals, which suffered from meningitis. In total, 21 GSs were significantly (FDR q ≤ 0.25) enriched. The majority of the GSs belong in the category of cellular signaling, with these GSs being TNFα signaling *via* NFκB, IL2-STAT5, TGFβ. The GS categories for immune system, cellular development, and cellular pathways all displayed an equal amount of enriched GSs. The inflammatory response, IL6-JAK/STAT3 signaling, and coagulation are categorized as immune response. The GSs angiogenesis, myogenesis, and EMT can be categorized under cellular development, and hypoxia, unfolded protein response, and reactive oxygen species pathway are categorized under cellular pathways.

**Table 5:** GSEA of the 21 hallmark GSs, which are significantly enriched in animals suffering from meningitis.

Hallmark Gene Set Name	Size	NES	FDR q-value
TNFα signaling *via* NFκB	181	2.77	0.000
Inflammatory response	168	1.98	0.001
IL6-JAK/STAT3 Signaling	69	1.82	0.006
IL2-STAT5 Signaling	174	1.79	0.006
TGFβ Signaling	49	1.73	0.008
Hypoxia	167	1.70	0.008
Estrogen response_early	168	1.58	0.020
Androgen response	89	1.52	0.029
Wnt/β-catenin	36	1.52	0.026
UV response_up	133	1.47	0.036
Myc targets	49	1.40	0.059
Angiogenesis	30	1.36	0.074
Cholesterol homeostasis	66	1.35	0.073
UV response_down	129	1.33	0.078
Coagulation	106	1.33	0.074
Estrogen response_late	167	1.33	0.071
Unfolded protein response	94	1.29	0.089
KRAS Signaling_up	174	1.25	0.118
Reactive oxigen species pathway	40	1.24	0.115
Myogenesis	165	1.19	0.172
Epithelial-Mesenchymal transition	178	1.17	0.186

The normalized enrichment score (NES) represents the extent of GS enrichment, taking the number of genes (“size”), which compose the GS, into account. The false discovery rate (FDR) is considered significant if q is ≤ 0.25.

NES, normalized enrichment score; FDR, false discovery rate.

**Figure 4:**
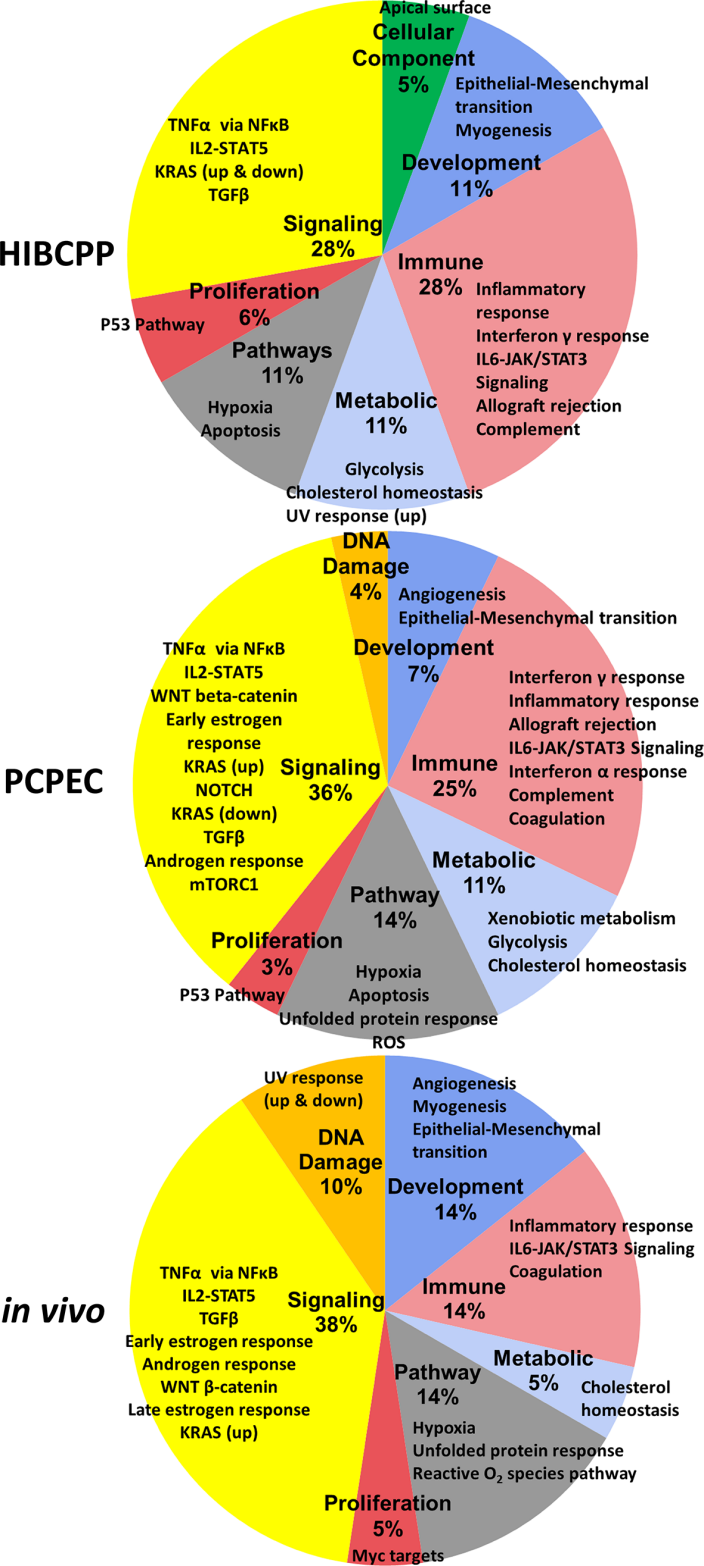
Summary of the enriched GSs of the hallmark GSEA for *S. suis* ST2 infected HIBCPP cells, PCPEC, and pigs. These pie-charts summarize the enriched GSs from **Table 4** to **Table 6**. The percentage, which is listed under the bold labels indicating the cellular process category, is based on the total number of significantly (FDR q ≤ 0.025) enriched GSs for the sample. The outer labels of the pie chart represent the significantly enriched GS for each sample which falls under the respective cellular process category.

A summary of the significantly enriched GSs designated to the respective cellular processes, as well as the percentage of the designated enriched GSs of the total number of significant GSs, are depicted in **Figure 4**.

GSs which are enriched in uninfected cells or meningitis-free animals in comparison to the infected or diseased state were also evaluated and are summarized in **Table 6**. These GSs display a negative NES score and a FDR q ≤ 0.25. In uninfected HIBCPP cells, the majority of enriched GSs were found to belong to the category of cellular proliferation. Furthermore, an enrichment of GSs belonging to the cellular categories of pathways, DNA damage, and development, was observed.

**Table 6:** The hallmark GSs, which are enriched in uninfected cells or meningitis-free animals.

Hallmark Gene Set Name	Size	NES	FDR q-value
uninfected HIBCPP cells			
MYC targets V2	58	−2.29	0.000
MYC targets V1	197	−1.90	0.000
E2F targets	197	−1.86	0.000
Unfolded protein response	109	−1.57	0.020
G2M Checkpoint	195	−1.56	0.016
DNA repair	138	−1.50	0.035
Spermatogenesis	64	−1.42	0.075
uninfected PCPEC			
E2F Targets	150	−1.54	0.128
Pancreas Beta Cells	18	−1.37	0.245
meningitis-free pigs			
Interferon α response	81	−2.57	0.000
Interferon γ response	163	−1.99	0.000
Oxidative phosphorylation	152	−1.72	0.003
E2F Targets	166	−1.62	0.010
DNA Repair	119	−1.40	0.122
Fatty Acid Metabolism	130	−1.38	0.122
Allograft Rejection	173	−1.32	0.186
Bile acid metabolism	97	−1.30	0.202

The normalized enrichment score (NES) represents the extent of GS enrichment, taking the number of genes (“size”), which compose the GS, into account. The false discovery rate (FDR) is considered significant if q is ≤0.25.

NES, normalized enrichment score; FDR, false discovery rate.

The uninfected PCPEC displayed the least amount of significant enriched GSs. Most notable was the GS containing genes regulated by the E2F transcription factors, belonging to the cellular proliferation category displayed a significant enrichment.

The majority of enriched GSs in uninfected cells or meningitis-free pigs are involved in the immune response and cellular metabolism. The interferon α and γ response, as well as the allograft rejection GSs are categorized under immune response, whereas the metabolic processes include oxidative phosphorylation, as well as fatty acid and bile acid metabolism.

## Discussion

In this study, the global transcriptome response of *in vitro S. suis*-infected HIBCPP cells and PCPEC, as well as the CP tissue of *in vivo* infected piglets with meningitis, was analyzed *via* RNA-seq. Subsequent to sequencing, the generated data was evaluated on the differential expression of individual genes and was also biologically interpreted by evaluating the enrichment of GSs *via* the GSEA between the infected *versus* uninfected phenotypes.

A previous transcriptome investigation, utilizing microarray technology, of apically *S. suis* ST2 infected PCPEC, which *in vivo* would reflect the state of infection following bacterial translocation into the CNS, revealed that the CP epithelial cells contributed to the inflammatory response *in vitro* (Schwerk et al., 2011). The basolateral infection of PCPEC in this current study, which *in vivo* would reflect the state before bacteria enter the CNS, induced a strong transcriptional response, with the majority of the identified significant DEGs being involved in inflammation as well. Part of the inflammatory response is the upregulation of cytokines and chemokines (including IL1β, TNFα, IL1α, CCL3L1, CXCL8, AMCF-II, CXCL2, CCL2, CCL11, CCL5, and IL6), many of which were also identified in the previous study of apically infected PCPEC (Schwerk et al., 2011). IL1β was found to be the strongest up-regulated gene and is known to be involved in many physiological functions, among others, being a potent inducer of inflammatory signaling expressed by many different cell types, including the CNS (Hewett et al., 2012). In previous studies evaluating the *S. suis*-dependent cytokine production, IL1β, along with other key inflammatory cytokines, such as IL6 and IL8, was found to be produced by porcine monocytes and polymorphonuclear lymphocytes in a whole-blood system in response to infection, as well as in human THP-1 monocytes (Segura et al., 2002; Segura et al., 2006). Furthermore, utilizing a murine *in vivo S. suis* infection model, the transcription of IL1β, TNFα, and CCL2 in the brain during cerebral inflammation was demonstrated *via in situ* hybridization (Dominguez-Punaro et al., 2007). In addition to the cytokine and chemokine upregulation observed in this present work, the inflammatory response regulators SLAMF7, RELB, NFKBIZ, NFKBIA, and ZC3H12A were identified, further underlining the strong inflammatory response elicited by PCPEC during to infection.

The transcriptome of HIBCPP cells was previously investigated following the *in vitro* infection with *N. meningitidis* from the basolateral cell side and demonstrated a strong inflammatory response, which included the production of cytokines and chemokines (Borkowski et al., 2014). Studies involving infection of other human cells with *S. suis*, such as HBMEC or human immune cells, mainly focused their research on the inflammatory response of host cells, too, or on bacterial survival and interaction with host cells (Charland et al., 2000; Segura et al., 2002; Liu et al., 2011b; Meijerink et al., 2012). Interestingly, in this present study, whereas PCPEC displayed a strong inflammatory response to infection, genes attributed to hypoxia were found to play a more prominent role during infection in HIBCPP cells (including ZFP36L1, MXI1, KLHL24, PNRC1, CDKN1C, and DUSP2).

In addition to the hypoxic response, it was interesting to observe the significant differential regulation of various RNA types, which are known to have a regulatory function and are non-protein coding. These regulatory RNAs included the anti-sense RNAs HLA-F, LIFR, PRKAR2A, ZEB1, and long intergenic non-coding RNAs (LINC) 887, 1431, 2482, as well as microRNAs (MIRs) 22HG and 3648, which are associated with viral inflammation and cancer and cellular proliferation, respectively (Rashid et al., 2017; Razooky et al., 2017; Zhang et al., 2018). This suggests that non-coding RNAs play a role in human CP epithelial cells during infection.

In this study, for the first time the transcriptome of cells found at the CP of pigs was investigated. For this sample set, it is important to consider that the CP tissue obtained from the *in vivo* infected pigs consists of a heterogeneous cell population, such as CP epithelial, endothelial cells, and immune cells located at or recruited to the BCSFB (Strazielle and Ghersi-Egea, 2000). Therefore, the results of the CP tissues samples cannot directly be compared to that of the *in vitro* infection experiments with homogenous PCPEC and HIBCPP epithelial cell populations. However, this data is rich in information, which helps to understand the CP tissue-specific response during *S. suis* infection. Previous studies carried out utilizing *in vivo* porcine *S. suis* infection models investigated the transcriptome of different organs (brain, lung, monocytes, spleen), but not specifically the CP (Li et al., 2010; Liu et al., 2011a). These studies evaluated the transcriptome at pre-defined time points (24 h and 3 days post-infection) of 4- to 5-week-old piglets, as compared to this present study, where the response of 8-week-old pigs was analyzed which developed acute meningitis, finding differentially regulated genes to be predominantly associated with the host’s inflammatory response.

The identification of biological pathways, in which multiple DEGs are connected to a common or networked performance, is of high interest in understanding cellular processes. Employing GSEA we identified GSs, which were significantly enriched in uninfected samples in comparison to infected samples. The majority of these GSs can be categorized into cellular metabolism or cellular proliferation. The enrichment of these GSs gives insight into metabolic alterations, which occur at the CP during *S. suis* infection, and is indicative that cellular proliferation is altered or stopped during *S. suis* ST2 infection. One enriched GS, which contains genes regulated by family members of E2F transcription factors, was found to overlap between all of the samples. The E2F transcription factors are important for the regulation of genes involved in DNA replication and the cell division cycle, and in the context of infection, they were previously investigated during viral infections, such as human immunodeficiency virus and adenovirus (Kundu et al., 1997; Bracken et al., 2004; Zheng et al., 2016).

A total of 28 GSs was identified to be significantly enriched in *S. suis*-infected PCPEC. The top enriched GSs are implicated in the immune response, complementing the data obtained in the DEG analysis. In a previous transcriptome study analyzing apically *S. suis*-infected PCPEC cells, Gene Ontology (GO) analysis, which evaluates high through-put data, based on biological processes, molecular functions, and cellular components (Ashburner et al., 2000), also identified many enriched cellular processes to be involved in inflammation, as revealed by the over-represented GO terms for cytokine activity, inflammatory response, defense response, and IκB kinase/NFκB cascade, as well as programmed cell death, which can be induced as a consequence of inflammation (Schwerk et al., 2011).

For the HIBCPP cell samples set, 18 GSs were found to be significantly enriched, with the most significant enrichment being for the GS containing genes in the hypoxia cellular stress response, thereby confirming its corresponding DEG analysis. In addition to the gene enrichment in hypoxia, the GSEA revealed an enrichment of GSs participating in inflammation. A previous GSEA and a GO analysis of *N. meningitidis*-infected HIBCPP cells revealed a predominant inflammatory response, with cytokine and chemokine activity playing a significant role (Borkowski et al., 2014). Additionally, a GS involved in wound healing was identified (Borkowski et al., 2014), which is often associated with epithelial–mesenchymal transition (EMT). The EMT GS was identified in the present study to be the third most enriched GS in infected HIBCPP cells. In a different study investigating the transcriptome of a human monocyte cell line exposed to *S. suis*, GO analysis revealed a cellular response including the participation of intracellular signaling pathways involved in inflammation, such as apoptosis and host defense and immunity, as well as other cellular processes involving cellular metabolism, gene transcription, and gene translation (Liu et al., 2011b).

The GSEA of the CP isolated from *in vivo*-infected piglets identified 21 significantly enriched GSs. The majority of the GSs displayed involvement in the inflammatory host response, as was also observed with the PCPEC samples. A previous porcine *in vivo* investigation revealed that the brains of *S. suis*-infected piglets displayed gene enrichment also involved in inflammation, and could be categorized into the GO terms of biological processes responding to a stimulus *via* signal transducer activity, cytokine activity and binding, defense response, inflammatory response, immune response, and innate immune response (Liu et al., 2011a). However, it was noted that many GO categories overlapped, due to the same genes being involved in multiple biological processes (Liu et al., 2011a). A further transcriptome study utilizing a zebrafish *S. suis* infection model, underscored the role of inflammation and host defense during infection (Wu et al., 2010).

**Figure 5:**
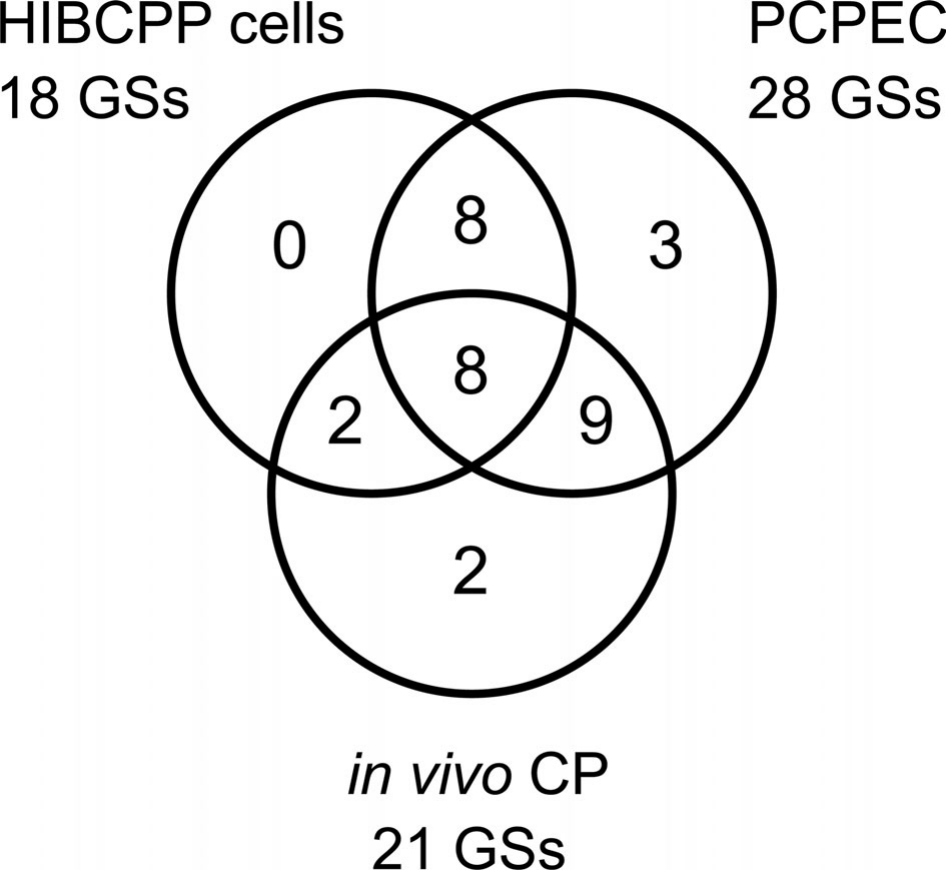
Overlap of the GSEA identified GSs for *S. suis* ST2 infected HIBCPP cells, PCPEC, and the CP of pigs suffering from *S. suis*-induced meningitis. The Venn diagram depicts the number of significantly enriched GSs attributed to each sample set with the number of GSs which were found to overlap between the infected epithelial cells (HIBCPP cells and PCPEC) and the epithelial cells with the *in vivo* CP tissue from meningitis cases, as well as the number of GSs overlapping between all three samples sets analyzed. Eight GSs were found to overlap in the three analyzed samples sets.

When comparing the 18, 28, and 21 significantly enriched GSs from *S. suis* ST2 infected HIBCPP cells, PCPEC, and animals suffering from meningitis, respectively, a total of eight GSs were found to overlap (**Figure 5**). These included the five GSs TNFα signaling *via* NFκB, inflammatory response, IL2-STAT5 signaling, IL6-JAK/STAT3 signaling, and TGFβ signaling, which were previously implicated during *S. suis* infection, whereas the three GSs for hypoxia, EMT, and a set of genes up-regulated by KRAS signaling, were not. Activation of the transcription factor NFκB regulates the expression of genes involved in the inflammatory response, including the expression of cytokines and chemokines, such as TNFα (Liu et al., 2017). An increase of TNFα, along with other pro-inflammatory cytokines, was detected in the brain, blood, and kidney of *S. suis*-infected mice, as well as in a porcine *in vivo* infection model, which investigated the brain, mononuclear cells, and lung (Liu et al., 2011a; Nakayama et al., 2011). Furthermore, the virulence associated factor suilysin of *S. suis* was shown to induce the release of TNFα in human monocytes (Lun et al., 2003) and, by utilizing PCPEC, microarray analysis following *S. suis* infection from the apical cell side revealed a strong induction of TNFα and other inflammatory cytokines (Schwerk et al., 2011). Interestingly, the release of TNFα was found to promote the permeability in a human BCSFB *in vitro* model, by inducing cell death (Schwerk et al., 2010). The release of cytokines is known to elicit a downstream cellular signaling cascade. One such signaling cascade is the IL6 induction of JAK/STAT3 signaling, which has been described to influence cell growth and differentiation, and is, therefore, often implicated in cancer when dysregulated (Huynh et al., 2017). Furthermore, as with IL6, IL2 has been described to stimulate the STAT5 signaling cascade, which is known to mainly regulate cellular proliferation and modulate the immune response of T cells (Lin and Leonard, 2000). In the context of *S. suis* infection, STAT3 and STAT5 were found to be up-regulated in the porcine brain *in vivo* (Liu et al., 2011a). However, the roles of these signaling cascades during *S. suis* infection have not been further investigated.

Another well described signaling cascade can be induced by the cytokine TGFβ, which was previously found to be upregulated in the porcine spleen and brain of *S. suis in vivo* infected piglets (Li et al., 2010; Liu et al., 2011a). The induction of TGFβ signaling was also implicated during the colonization of *Streptococcus pneumoniae* and *Haemophilus influenzae*, which are other known bacterial meningitis-causative agents, in lung epithelial cells and was accompanied by an inflammatory response (Beisswenger et al., 2009).

To date, hypoxia, EMT, and genes regulated by KRAS signaling have not been directly linked to *S. suis* infection. It should be considered that an enrichment of the KRAS GS in this present study was observed due to overlapping genes found with the GS EMT, which is a cellular program that reverts epithelial cells to mesenchymal cells and is often implicated in cancer, since it promotes metastasis and invasion (Kalluri and Weinberg, 2009). EMT is also often involved in wound healing and tissue regeneration, which occurs following a physical trauma or injury caused by inflammation as a result of, e.g., an infection (Kalluri and Weinberg, 2009; Hofman and Vouret-Craviari, 2012). Furthermore, previous studies, especially in cancer research, have demonstrated that a hypoxic environment is a prerequisite in order for EMT to occur (Cannito et al., 2008).

A hypoxic microenvironment is often associated with infected tissues and elicits a stress response due to oxygen being a necessity in order for animal cells to carry out their normal physiological functions (Taylor and Colgan, 2017). Hypoxia has been implicated during injury to the CNS, which includes stroke or infection (van der Flier et al., 2003; Mukandala et al., 2016) and affects the global transcription and translation of mRNA molecules by reducing both of these molecular processes, which allows for energy conservation during cellular stress (Koritzinsky and Wouters, 2007). Since the hypoxia GS was the top significantly enriched GS in the infected HIBCPP samples, it could be speculated as a reason for why the HIBCPP cells did not exhibit a strong fold change in the DEG analysis, as compared to the PCPEC post-infection.

Hypoxia stress response can be induced either in a hypoxia-inducible factor (HIF) dependent or independent manner, most notably *via* the NFκB transcription factor, with complex overlaps and cross-talks described between these two pathways (Rius et al., 2008; Schaffer and Taylor, 2015). NFκB activation is known to be associated with the acute phase of hypoxia, as well as cytokine release, most notable being IL1β and TNFα (Mukandala et al., 2016). Additionally, IL1β and TNFα can activate the HIF transcription factor in an oxygen-independent and NFκB-dependent pathway during inflammation (Lin and Simon, 2016). Interestingly, the inflammatory cytokine IL1β was observed to affect BBB permeability by influencing blood vessel plasticity *via* HIF regulation in the angiogenesis program in multiple sclerosis patients (Argaw et al., 2006). A further study investigating the cytokine presence in the CSF of infants who suffered a perinatal hypoxia event, found that infants who displayed an elevated amount of TNFα and IL6 in the CSF, were significantly more likely to suffer from severe neurological abnormalities 12 months post-partum; a phenomenon, which was also observed in survivors of bacterial meningitis and cerebral malaria (John et al., 2008; Perdomo-Celis et al., 2015; Sumanovic-Glamuzina et al., 2017).

Hypoxia does not only induce a cellular stress response, but is also described to modulate cell functions, e.g. of epithelial cells during infection (Schaffer and Taylor, 2015; Taylor and Colgan, 2017). In two previous studies, it was demonstrated that a hypoxic environment during the infection with *Pseudomonas aeruginosa* of lung epithelial cells reduced the host’s cellular uptake of the pathogen, thereby diminishing host cell death, as well as causing a decline in the invasion of *Yersinia enterocolitica* into intestinal epithelial cells *in vitro* (Schaible et al., 2013; Zeitouni et al., 2016). Furthermore, hypoxia also negatively affected the virulence factor expression (Schaible et al., 2017). These studies demonstrate that a hypoxic environment was able to aid in containing the infection.

Here, we observed to our knowledge for the first time, that hypoxia-related cellular processes were significantly increased in relation to *S. suis* ST2 infection *in vitro* with human CP epithelial cells and PCPEC, as well as in the *in vivo* infected porcine CP samples. One gene in particular stood out in the enriched hypoxia GS in all three analyzed samples sets. VEGF was found to be one of the top enriched genes in the hypoxia GS of all analyzed samples (data not shown), which is indicative of angiogenesis, and in turn has been implicated in chronic hypoxia response (Mukandala et al., 2016).

The hypoxia transcription factor HIF has been discussed as a potential target for adjunctive therapy options, with already successful candidates against methicillin-sensitive and -resistant *Staphylococcus aureus* strains, in order to improve outcome or potentially replace antimicrobial therapeutics due to a rise in microbial resistance (Zinkernagel et al., 2008; Okumura et al., 2012; Santos and Andrade, 2017). Importantly, HIF therapy would not be solely limited to infections, but could also be applied in the setting of cancer treatment or chronic inflammatory disorders (Santos and Andrade, 2017). By investigating the role of hypoxia in the context of *S. suis* infection, with a focus on the significance of the BCSFB, a long-term aim could be the exploration of alternative therapy options. Those alternative therapy options could potentially limit infection severity, which is often found corresponding to long-term sequelae of survivors, such as full or partial auditory loss frequently associated with *S. suis* infection in humans, or death.

## Data Availability Statement

The datasets presented in this study can be found in online repositories. The names of the repository/repositories and accession number(s) can be found below: https://www.ncbi.nlm.nih.gov/, PRJNA533919; https://www.ncbi.nlm.nih.gov/, PRJNA533792; https://www.ncbi.nlm.nih.gov/, PRJNA534398.

## Ethics Statement

In vivo piglet infection experiments, and the subsequent necropsy, were carried out by veterinarians, in compliance with the principles outlined in the European Convention for the Protection of Vertebrate Animals Used for Experimental and Other Scientific Purposes, as well as the German Animal Protection Law (Tierschutzgesetz). The CP tissue samples analyzed in this study originated from two different *in vivo* infection studies. The CP tissue samples from the meningitis-free animals were part of a study, which was approved by the Landesdirektion Sachsen, with the permit number TVV28/16, which includes approval through the registered committee for animal experiments. The experiment analyzing the CP from animals suffering from meningitis were approved by the Committee on Animal Experiments of the Lower Saxonian State Office for Consumer Protection and Food Safety under the permit number 33.12-42,502-04-16/2305A (Rungelrath et al., 2018). The piglets of both studies originated from the same German Landrace herd that is based on the genotyping results of more than 400 S. suis isolates free of the S. suis pathotype investigated in this study.

## Author Contributions

AL, HS, and CS conceived and coordinated the study. AL performed underlying experiments. AB and KK performed histology. CB and PV-W coordinated animal experiments. RS and LK-H performed RNA-seq analysis. AL, RS, and LK-H performed the statistics. CB, PV-W, and HI provided resources. AL, CS, and HS drafted the manuscript. All authors contributed to the article and approved the submitted version.

## Funding

Partial financial support of this research was awarded by the Grimminger-Stiftung für Zoonoseforschung (Grimminger Foundation for Zoonotic Research). The animal experiments were financially supported by a grant of the German Research Foundation (DFG BA 4730/3-1) to CB.

## Conflict of Interest

The authors declare that the research was conducted in the absence of any commercial or financial relationships that could be construed as a potential conflict of interest.
