# Supplementary material for: A Comparative Transcriptome Analysis of Human and Porcine Choroid Plexus Cells in Response to Streptococcus suis Serotype 2 Infection Points to a Role of Hypoxia
Source: Front Cell Infect Microbiol. 2021 Mar 8;11:639620. doi: 10.3389/fcimb.2021.639620 (PMC7982935; doi:10.3389/fcimb.2021.639620)
Supplement: Supplementary file 1 [file DataSheet_1.pdf]

Supplementary Table S1. Oligonucleotide primers used for the RT-PCR.

Gene	Oligonucleotide primer for RT-PCR	Annealing Temp. (℃)	Amplicon size (bp)	Reference
**Human**				
gapdh	Forward 5'-TGTTGCCATCAATGACCCCTT-3'<br>Reverse 5'-CTCCACGACGTACTCAGCG-3'	60	202	Borkowski et al. (2014)
nfkbiz	Forward 5'-CAGTTCAAGTTAGCTGGCTGA-3'<br>Reverse 5'-TCTGTGGAGAATACTGGTACAGG-3'	60	177	Borkowski et al. (2014)
il8	Forward 5'-CAAGAGCCAGGAAGAAACCA-3'<br>Reverse 5'-GTCCACTCTCAATCACTCTCAG-3'	60	225	Borkowski et al. (2014)
tnf-α	Forward 5'-GAGCACTGAAAGCATGATCC-3'<br>Reverse 5'-CGAGAAGATGATCTGACTGCC-3'	60	234	Borkowski et al. (2014)
cxcl1	Forward 5'-CTCTTCCGCTCCTCTCACAG-3'<br>Reverse 5'-GGGGACTTCACGTTCACACT-3'	60	239	van Sorge et al. (2008)
cxcl2	Forward 5'-CTCAAGAATGGGCAGAAAGC-3'<br>Reverse 5'-AAACACATTAGGCGCAATCC-3'	60	213	van Sorge et al. (2008)
**Porcine**				
gapdh	Forward 5'-CAGCCTCAAGATCATCAGCA-3'<br>Reverse 5'-TCCAGGGGCTCTTACTCCTT-3'	55	594	Tenenbaum et al. (2008)
il8	Forward 5'-GATGCCAACACAACTTCAATCA-3'<br>Reverse 5'-GCCAAAACAGGATTTCCAGC-3'	60	221	This study
il1β	Forward 5'-ATGCCAACGTGCAGTCTATG-3'<br>Reverse 5'-GAAGACGGGCTTTTGTTCTG-3'	55	400	This study
cxcl2	Forward 5'-ACTGCTCTTGTTTAGCATCTTCTC-3'<br>Reverse 5'-TGACCAAACGGAAGTCATAGC-3'	55	109	This study

bp, base pairs; *cxcl*, (C-X-C motif) ligand; *gapdh*, glyceraldehyde-3-phosphate dehydrogenase; *il*, interleukin; *nfkbiz*, NF-kappa-B inhibitor zeta; *tnf-α*, tumor necrosis factor alpha

References

Borkowski, J., Li, L., Steinmann, U., Quednau, N., Stump-Guthier, C., Weiss, C., Findeisen, P., Gretz, N., Ishikawa, H., Tenenbaum, T.*, et al.* (2014). Neisseria meningitidis elicits a pro-inflammatory response involving IkappaBzeta in a human blood-cerebrospinal fluid barrier model. J. Neuroinflammation *11*, 163.

Tenenbaum, T., Matalon, D., Adam, R., Seibt, A., Wewer, C., Schwerk, C., Galla, H.J., and Schroten, H. (2008). Dexamethasone prevents alteration of tight junction-associated proteins and barrier function in porcine choroid plexus epithelial cells after infection with Streptococcus suis in vitro. Brain Res. *1229*, 1-17.

van Sorge, N.M., Ebrahimi, C.M., McGillivray, S.M., Quach, D., Sabet, M., Guiney, D.G., and Doran, K.S. (2008). Anthrax toxins inhibit neutrophil signaling pathways in brain endothelium and contribute to the pathogenesis of meningitis. PLoS One *3*, e2964.

Supplementary Table S2. Oligonucleotide primers used for the QPCR.

Gene	Oligonucleotide primer for QPCR	Annealing Temp. (℃)	Amplicon size (bp)	Reference
**Human**				
gapdh	Forward 5'-TGTTGCCATCAATGACCCCTT-3'	60	202	(Borkowski et al., 2014)
	Reverse 5'-CTCCACGACGTACTCAGCG-3'			
il1β	Forward 5'-AGCTACGAATCTCCGACCAC-3'	60	186	This study
	Reverse 5'-CGTTATCCCATGTGTCGAAGAA-3'			
il8	Forward 5'-CAAGAGCCAGGAAGAAACCA-3'	60	225	(Borkowski et al., 2014)
	Reverse 5'-GTCCACTCTCAATCACTCTCAG-3'			
cxcl2	Forward 5'-CTCAAGAATGGGCAGAAAGC-3'	60	213	(van Sorge et al., 2008)
	Reverse 5'-AAACACATTAGGCGCAATCC-3'			
tnf-α	Forward 5'-GAGCACTGAAAGCATGATCC-3'	60	234	(Borkowski et al., 2014)
	Reverse 5'-CGAGAAGATGATCTGACTGCC-3'			
nfkbia	Forward 5'-CTCCGAGACTTTCGAGGAAATAC-3'	60	135	This study
	Reverse 5'-GCCATTGTAGTTGGTAGCCTTCA-3'			
zc3h12a	Forward 5'-GGCAGTGAACTGGTTTCTGGA-3'	60	232	(Borkowski et al., 2014)
	Reverse 5'-GATCCCGTCAGACTCGTAGG-3'			
tcim	Forward 5'-AGAAGAGCCTGGACAGATGT-3'	58	175	This study
	Reverse 5'-TTTTCTCTCCCTGTCCTCCC-3'			
hif1α	Forward 5'-GAACCCATTCCTCACCCATC-3'	58	250	This study
	Reverse 5'-GCATCCTGTACTGTCCTGTG-3'			
vegfa	Forward 5'-TCTTCAAGCCATCCTGTGTG-3'	58	235	This study
	Reverse 5'-CCCTTTCCCTTTCCTCGAAC-3'			
mxi1	Forward 5'-CTCTCTTCCTCACAGTCCCA-3'	58	194	This study
	Reverse 5'-CAGGGGTAAGGTCTCCAAGA-3'			
dusp2	Forward 5'-ATCTTGCCCTACCTGTTCCT-3'	58	222	This study
	Reverse 5'-GCTGTTCTTCACCCAGTCAA-3'			
**Porcine**				
Gapdh	Forward 5'-TGGTGCTACGTATGTTGTGG-3'	58	177	This study
	Reverse 5'-ATTGCTGACGATCTTGAGGG-3'			
il1β	Forward 5'-GAGTGCAAACTCCAGGACAA-3'	58	218	This study
	Reverse 5'-GGTGGGCGTGTTATCTTTCA-3'			
il8	Forward 5'-GATGCCAACACAACTTCAATCA-3'	60	221	This study
	Reverse 5'-GCCAAAACAGGATTTCCAGC-3'			
cxcl2	Forward 5'-ACTGCTCTTGTTTAGCATCTTCTC-3'	55	109	This study
	Reverse 5'-TGACCAAACGGAAGTCATAGC-3'			
tnfα	Forward 5'-ACGTTTTCCTCACTCACACC-3'	58	176	This study
	Reverse 5'-TCGATCATCCTTCTCCAGCT-3'			
nfkbia	Forward 5'-TGTCGCTCTTGTTGAAGTGT-3'	58	193	This study
	Reverse 5'-TCTGTGAACTCTGACTCCGT-3'			
zc3h12a	Forward 5'-CTGTAGGATTGGTTCTGGCC-3'	58	156	This study
	Reverse 5'-CGAGAGAAAGGAGGGTTTGG-3'			
tcim	Forward 5'-CCGATCAACTTCACGTCTGT-3'	58	181	This study
	Reverse 5'-CTGCAATTCTTCTCCAGCCT-3'			
hif1α	Forward 5'-TGTAATGCTCCCCTCATCCA-3'	58	198	This study
	Reverse 5'-GGCTGATCTTGAATCTGGGG-3'			
vegfa	Forward 5'-CGAGTACATCTTCAAGCCGT-3'	58	154	This study
	Reverse 5'- CATCTCTCCTATGTGCTGGC-3'			
mxi1	Forward 5'- GCAGAGTATTGGGAGTGACG-3'	58	155	This study
	Reverse 5'-GAGACTGCAACATGAACCCA-3'			
dusp2	Forward 5'-CAGATGGTGGAGATCAGTGC-3'	58	228	This study
	Reverse 5'-CTGCCCCATGAAACTGAAGT-3'			

dusp2, dual specificity phosphatase 2; *hif1α*, hypoxia-inducible factor 1 alpaha; *mxi*, MAX interactor 1; *nfkbia*, NFαB inhibitor alpha; *tcim*, transcriptional and immune response regulator; *vegfα*, vascular endothelial growth factor alpha; *zc3h12a*, zinc finger CCCH-type containing 12A

References

Borkowski, J., Li, L., Steinmann, U., Quednau, N., Stump-Guthier, C., Weiss, C., Findeisen, P., Gretz, N., Ishikawa, H., Tenenbaum, T.*, et al.* (2014). Neisseria meningitidis elicits a pro-inflammatory response involving I kappa B zeta in a human blood-cerebrospinal fluid barrier model. J. Neuroinflamm *11*, 163.

van Sorge, N.M., Ebrahimi, C.M., McGillivray, S.M., Quach, D., Sabet, M., Guiney, D.G., and Doran, K.S. (2008). Anthrax toxins inhibit neutrophil signaling pathways in brain endothelium and contribute to the pathogenesis of meningitis. PLoS One *3*, e2964.

Supplementary Table S3. A total of 63 genes were significantly differentially expressed in *S. suis* ST2 infected versus uninfected HIBCPP cells. The significance and gene fold change were determined with the PartekGS software implementing the one-way ANOVA test. Significant DEGs with a ±2 fold change and an uncorrected *p*-value ≤ 0.05 are presented.

Gene Symbol	Gene Name	*p*-value	Fold Change
ZFP36L1	ZFP36 ring finger protein like 1	0.0427	3.6
PCK1	Phosphoenolpyruvate carboxykinase 1	0.0254	3.3
TCIM	Transcriptional and immune response regulator	0.0249	3.1
LOC100996419	uncharacterized ncRNA	0.0247	3.0
C8orf44-SGK3	Chromosome 8 open reading frame 44 - Serum/glucocorticoid regulated kinase family member 3 readthrough	0.0415	2.9
HLA-F-AS1	Major histocompatibility complex, class I, F antisense RNA 1	0.0059	2.7
ARIH2OS	Ariadne RBR E3 ubiquitin protein ligase 2 opposite strand	0.0420	2.7
FSBP	Fibrinogen silencer binding protein	0.0223	2.6
FBXL8	F-box and leucine rich repeat protein 8	0.0137	2.6
LIFR-AS1	Leukemia inhibitory factor receptor antisense RNA1	0.0373	2.5
RASA4	RAS p21 protein activator 4	0.0090	2.5
MXI1	MAX interactor 1, dimerization protein	0.0430	2.5
LINC00887	Long intergenic non-protein coding RNA 887	0.0136	2.5
MHENCR	Melanoma highly expressed competing endogenous lncRNA for miR-425 and miR489	0.0373	2.4
ARL14	ADP ribosylation factor like GTPase 14	0.0211	2.4
PIK3IP1	Phosphoinositide-3-kinase interacting protein 1	0.0063	2.4
LINC01431	Long intergenic non-protein coding RNA 1431	0.0484	2.3
LINC02482	Long intergenic non-protein coding RNA 2482	0.0027	2.3
C12orf76	Chromosome 12 open reading frame 76	0.0302	2.3
KLHL24	Kelch like family member 24	0.0091	2.3
RND1	Rho family GTPase 1	0.0282	2.2
TCP11L2	T-complex 11 like 2	0.0372	2.2
PNRC1	Proline rich nuclear receptor coactivator 1	0.0395	2.1
PRKAR2A-AS1	Protein kinase cAMP-dependent type II regulatory subunit alpha antisense RNA 1	0.0101	2.1
CDKN1C	Cyclin dependent kinase inhibitor 1C	0.0288	2.0
RNF39	Ring finger protein 39	0.0149	2.0
FHOD3	Formin homology 2 domain containing 3	0.0349	2.0
ZEB1-AS1	Zinc finger E-box binding homeobox 1 antisense RNA 1	0.0122	2.0
DBP	D-box binding PAR bZIP transcription factor	0.0052	2.0
LOC440934	uncharacterized ncRNA	0.0230	2.0
LENG8-AS1	Leukocyte receptor cluster, member 8 antisense RNA 1	0.0325	2.0
MST1	Macrophage stimulating 1	0.0073	2.0
LCMT2	Leucine carboxyl methyltransferase 2	0.0025	-2.0
SHOX2	Short stature homeobox 2	0.0091	-2.0
TRIM25	Tripartite motif containing 25	0.0236	-2.0
IBA57	IBA57 homolog, iron-sulfur cluster assembly	0.0043	-2.0
URB2	URB2 ribosome biogenesis homolog	0.0177	-2.0
ZNF778	Zinc finger protein 778	0.0263	-2.0
RGMB	Repulsive guidance molecular family member b	0.0153	-2.0
CLN8	Transmembrane endoplasmic reticulum (ER) and ER-Golgi Intermediate Compartment protein	0.0137	-2.1
CD3EAP	CD3e molecule associated protein	0.0122	-2.1
NBPF12	Neuroblastoma breakpoint family member 12	0.0084	-2.1
SLC46A1	Solute carrier family 46 member 1	0.0013	-2.1
CYB561D1	Cytochrome b561 family member D1	0.0469	-2.1
EN2	Engrailed homeobox 2	0.0278	-2.1
CEBPA	CCAAT enhancer binding protein alpha	0.0105	-2.1
DHRS9	Dehydrogenase reductase 9	0.0379	-2.2
CHAC2	ChaC cation transport regulator homolog 2	0.0084	-2.2
FBXO9	F-box protein 9	0.0280	-2.2
ADAM1A	ADAM metallopeptidase domain 1A	0.0321	-2.3
ZNF283	Zinc finger protein 283	0.0147	-2.3
MIR22HG	lncRNA MIR22 host gene	0.0424	-2.4
LRRC26	Leucine rich repeat containing 26	0.0158	-2.4
POC1B-GALNT4	POC1 centriolar protein B - Polypeptide N-acetylgalactosaminyltransferase 4 readthrough	0.0159	-2.5
EIF5	Eukaryotic translation initiation factor 4E	0.0293	-2.6
DUSP2	Dual specificity phosphatase 2	0.0146	-2.6
CDK5R1	Cyclin dependent kinase 5 regulatory subunit 1	0.0145	-2.8
FGF18	Fibroblast growth factor 18	0.0347	-2.8
KBTBD8	Kelch repeat and BTB domain containing 8	0.0338	-3.1

Supplementary Table S4. A total of 50 genes were significantly differentially expressed in *S. suis* ST2 infected versus uninfected PCPEC. The significance and gene fold change were determined with the PartekGS software implementing the one-way ANOVA test. Significant DEGs with a ±2 fold change and an uncorrected *p*-value ≤ 0.05 are presented.

Gene Symbol	Gene Name	*p*-value	Fold Change
IL1β	Interleukin 1 beta	0.041	134.0
TNF	tumor necrosis factor	0.006	70.7
ACOD1	aconitate decarboxylase 1	0.000	52.2
ENSSSCG00000032343	uncharacterized	0.001	39.3
IL1α	interleukin 1 alpha	0.002	29.3
CCL3L1	Chemokine (C-C motif) ligand 3-like 1	0.006	27.9
CXCL8	C-X-C motif chemokine ligand 8 (aka IL8)	0.006	22.6
AMCF-II	alveolar macrophage-derived chemotactic factor II (aka CXCL6)	0.009	15.1
CXCL2	chemokine (C-X-C motif) ligand 2	0.017	13.8
CCL2	chemokine (C-C motif) ligand 2	0.026	12.1
SLAMF7	SLAM family member 7	0.025	11.4
RND1	Rho family GTPase 1	0.023	10.9
ENSSSCG00000008954	uncharacterized	0.006	10.4
CCL11	chemokine (C-C motif) ligand 11	0.008	9.1
VCAM1	vascular cell adhesion molecule 1	0.019	8.0
PLEK	pleckstrin	0.003	5.1
DEPP1	DEPP1 autophagy regulator	0.009	5.0
RELB	avian reticuloendotheliosis viral (v-rel) oncogene related B	0.005	4.6
NFKBIZ	nuclear factor of kappa light polypeptide gene enhancer in B cells inhibitor, zeta	0.041	4.5
NFKBIA	nuclear factor of kappa light polypeptide gene enhancer in B cells inhibitor, alpha	0.016	3.9
CEBPD	CCAAT/enhancer binding protein (C/EBP), delta	0.027	3.2
GDF15	growth differentiation factor 15	0.001	2.8
IRF1	interferon regulatory factor 1	0.017	2.7
MAFF	v-maf musculoaponeurotic fibrosarcoma oncogene family, protein F (avian)	0.001	2.7
TCIM	Transcriptional and immune response regulator	0.001	2.7
SIK1	salt inducible kinase 1	0.000	2.6
CABP1	calcium binding protein 1	0.007	2.4
CCL5	chemokine (C-C motif) ligand 5	0.031	2.4
ICAM1	intercellular adhesion molecule 1	0.005	2.4
JUNB	jun B proto-oncogene	0.004	2.4
MAP3K8	mitogen-activated protein kinase kinase kinase 8	0.015	2.4
RSAD2	radical S-adenosyl methionine domain containing 2	0.034	2.3
STC2	stanniocalcin 2	0.036	2.3
PLAU	plasminogen activator, urokinase	0.020	2.3
PLEKHS1	pleckstrin homology domain containing, family S member 1	0.006	2.3
NEURL3	neuralized E3 ubiquitin protein ligase 3	0.015	2.3
SLCO1B3	solute carrier organic anion transporter family member 1B3	0.012	2.2
PDE4B	phosphodiesterase 4B, cAMP specific	0.001	2.2
IL6	interleukin 6	0.026	2.2
ELF3	E74-like factor 3	0.036	2.2
PFKFB3	6-phosphofructo-2-kinase/fructose-2,6-biphosphatase 3	0.003	2.1
SRGN	serglycin	0.050	2.1
ZC3H12A	zinc finger CCCH type containing 12A	0.004	2.1
FAM83D	family with sequence similarity 83, member D	0.005	2.0
TIFA	TRAF-interacting protein with forkhead-associated domain	0.023	2.0
KCNK3	potassium channel, subfamily K, member 3	0.020	2.0
VSIG4	V-set and immunoglobulin domain containing 4	0.017	-2.1
DLGAP5	DLG associated protein 5	0.046	-2.6
KNL1	kinetochore scaffold 1	0.029	-2.7
STRC	stereocilin	0.037	-3.0

Supplementary Table S5. A total of 30 genes were found to be significant Differentially Expressed Genes in the choroid plexus of pigs suffering from *S. suis* ST2-induced meningitis versus meningitis-free pigs. The significance and gene fold change were determined with the PartekGS software implementing the one-way ANOVA test. Significant DEGs with a ±2 fold change and a corrected *p*-value ≤ 0.05 are presented.

Gene Symbol	Gene Name	p-value	Fold Change
INSM1	insulinoma-associated 1	0.0002	11.3
GABRB1	gamma-aminobutyric acid A receptor, subunit beta 1	0.0002	5.0
INHBB	inhibin beta-B	0.0003	5.0
ENSSSCG00000000659	uncharacterized	0.0001	4.9
PVR	poliovirus receptor	0.0003	3.8
CUBN	cubilin	0.0001	2.9
EMP2	epithelial membrane protein 2	0.0003	2.7
CRNN	cornulin	0.0002	2.7
DERL3	Der1-like domain family, member 3	0.0002	2.7
HSP90B1	heat shock protein 90 kDa beta member 1	0.0000	2.2
GPR3	G-protein coupled receptor 3	0.0004	2.2
GPR146	G-protein coupled receptor 146	0.0004	2.0
ALDH3B1	aldehyde dehydrogenase 3 family member B1	0.0002	-2.0
DNAAF1	dynein, axonemal assembly factor 1	0.0002	-2.2
ENSSSCG00000036652	uncharacterized	0.0003	-2.3
HYKK	hydroxylysine kinase 1	0.0001	-2.3
MTMR8	myotubularin related protein 8	0.0003	-2.3
EIF2D	eukaryotic translation initiation factor 2D	0.0002	-2.4
SYTL1	synaptotagmin-like 1	0.0000	-2.5
POLE	polymerase (DNA directed), epsilon	0.0004	-2.5
ENSSSCG00000013144	uncharacterized	0.0000	-2.9
SORD	sorbitol dehydrogenase	0.0003	-2.9
VWA5A	von Willebrand factor A domain containing 5A	0.0002	-3.1
PRDM16	PR domain containing 16	0.0002	-3.3
RBM3	RNA binding motif protein 3	0.0002	-3.9
FLRT1	fibronectin leucine rich transmembrane protein 1	0.0004	-4.8
ACEA_U3		0.0001	-5.5
U3		0.0001	-5.5
TYMS	thymidylate synthase	0.0002	-6.1
TIMD4	T cell immunoglobulin and mucin domain containing 4	0.0002	-6.3
